# Parental folate deficiency induces birth defects in mice accompanied with increased de novo mutations

**DOI:** 10.1038/s41421-021-00364-0

**Published:** 2022-02-22

**Authors:** Ying Zhao, Duoyuan Chen, Jianping Tang, Yufang Zheng, Ji Qi, Hongyan Wang

**Affiliations:** 1 Obstetrics and Gynecology Hospital, Institute of Reproduction and Development, Fudan University, Shanghai, China; 2 State Key Laboratory of Genetic Engineering, School of Life Sciences, Fudan University, Shanghai, China; 3 Shanghai Laboratory Animal Research Center, Shanghai, China; 4 Sino-British SIPPR/B&K Lab Animal Ltd, Shanghai, China; 5 Department of Cellular and Developmental Biology, School of Life Sciences, Fudan University, Shanghai, China; 6 Children’s Hospital of Fudan University, 399 Wanyuan Road, Shanghai, China

**Keywords:** Molecular biology, Mechanisms of disease

## Abstract

Dietary folate deficiency (FD) is associated with the occurrence of birth defects. However, the mechanisms underlying this association remain elusive. In particular, how FD affects genome stability is unknown. To examine whether a folate-deficient diet can affect genome stability, C57BL/6 mice were maintained on a synthetic diet lacking of folic acid (FA) for two generations. F0 mice received the FD diet beginning at 3 weeks of age, and their offspring (F1) began the FD diet after weaning. Both male and female F1 mice fed the FD diet were intentionally crossed with F1 mice fed the normal diet to produce F2 mice. F2 embryos were dissected and collected at E14.5 and E18.5. The malformation ratio was significantly increased in F2 embryos fed the FD diet for two generations compared to those fed the normal diet. Whole-genome sequencing of multiple sibship with F1 males on the FD diet showed that the de novo mutation (DNM) rate in F2 embryos was three times of the reported spontaneous rate in mice. Furthermore, many DNMs observed in the F2 mice exhibited an allele ratio of 1:3 instead of 2:2, suggesting that these mutations are likely to accumulate in gamete cells as a form of mismatch in the DNA duplex. Our study indicated that FD for two generations significantly enhances DNM accumulation during meiosis, which might contribute to the increased negative birth outcomes among F2 mice. Not only maternal but also paternal FA supplementation is probably also necessary and beneficial to prevent birth defects.

## Introduction

Dietary folate is an essential micronutrient for life. It is required not only for epigenetic regulation of gene expression through methylation, but also for cell proliferation, as it is involved in de novo nucleotide synthesis. It has been shown that gestational folate deficiency (FD) is associated with poor reproductive outcomes and birth defects^1^, especially neural tube defects (NTDs)^2,3^, orofacial clefts^4,5^, and congenital heart diseases (CHDs)^2,6^. Periconceptional and early pregnancy folic acid (FA) supplementation has been recommended in many countries and has been shown to reduce the incidence of NTDs by ~20%–80%^7,8^ and that of CHDs by 31% in China^9^ and 11% in Canada^10^.

However, the mechanism by which FD causes an increased rate of birth defects is still unclear. It is also uncertain whether paternal folate supplementation can contribute to birth defect prevention. Since folate metabolism generates the major methyl donor SAM, most past studies have focused on epigenetic deregulation caused by FD. For example, maternal FD diets cause hypermethylation of the *Brachyury* gene and, in turn, downregulation of the FGF pathway in human NTD samples^11^, and paternal lifetime FD causes decreased sperm counts and alters the methylation of imprinted genes, such as *H19*, in BALB/c mice^12^. In addition to affecting methylation, it has been proposed that FD can also affect DNA stability/mutations, as folate metabolism is also linked to de novo purine and pyrimidine synthesis. However, to date, very few studies have focused on the effects of FD diets on chromosomal damage, and the results are controversial, with some studies indicating that they are effective^13,14^ and others indicating that they are not^15^. Currently, most studies have focused on the effect of maternal FD, mainly considering maternal impairment in terms of developing embryos or fetuses. It has been shown that maternal FD in mice causes not only increased fetal death and lower fetal weight, but also more septum defects in the heart^1,16^ and more craniofacial structure defects^4,5^. Maternal FD might have comprehensive effects on not only embryo development but also gamete formation; therefore, it is very difficult to assess the effect of FD on fetal genome stability. Comparatively, the effects of paternal FD on offspring are simpler, as this can be transferred to the offspring only via sperm. Only a few studies have studied the effects of parental FD. Previous studies have reported increased negative reproductive outcomes after paternal FD exposure^12,17^; however, the effects of a paternal FD diet and how it affects birth defects are still not clear.

Recent studies have shown that close to 80% of de novo mutations (DNMs) arise in the paternal lineage and that the number of DNMs increase with the age of the father^18–20^. DNMs are associated with an increased prevalence of several conditions including birth defects, intellectual disability, and autism spectrum disorders^18–21^. This inspired us to investigate whether paternal FD plays any role in increasing DNMs in germline cells to induce birth defects in offspring.

In our study, we used C57BL/6 mice to study DNA mutations caused by FD in two continuous generations. Mice were maintained on a FD diet for two generations, and F2 embryos were dissected and collected at E14.5 and E18.5. We analyzed the malformation ratio in F2 embryos and performed whole-genome sequencing (WGS) on collected samples from F0, F1, and F2 mice. Our results showed that the number of DNMs was significantly increased in F2 embryos from F1 male fed an FD diet with just one set of grandparents fed the FD diet. When both grandparents were on the FD diet, the number of DNMs was two times greater in F2 embryos than when one grandparent was on the FD diet. We also found that nearly one-quarter of DNMs in F1 mice and half of DNMs in F2 embryos exhibited an allele ratio of 1:3 instead of 2:2, this result suggested that those DNMs with allele ratio at 1:3 are likely came from mutations accumulated in gamete cells. To our knowledge, this is the first effort to study the effect of a FD diet on DNMs in germline cells.

## Results

### FD for two generations increased malformations among F2 embryos

**Fig. 1:**
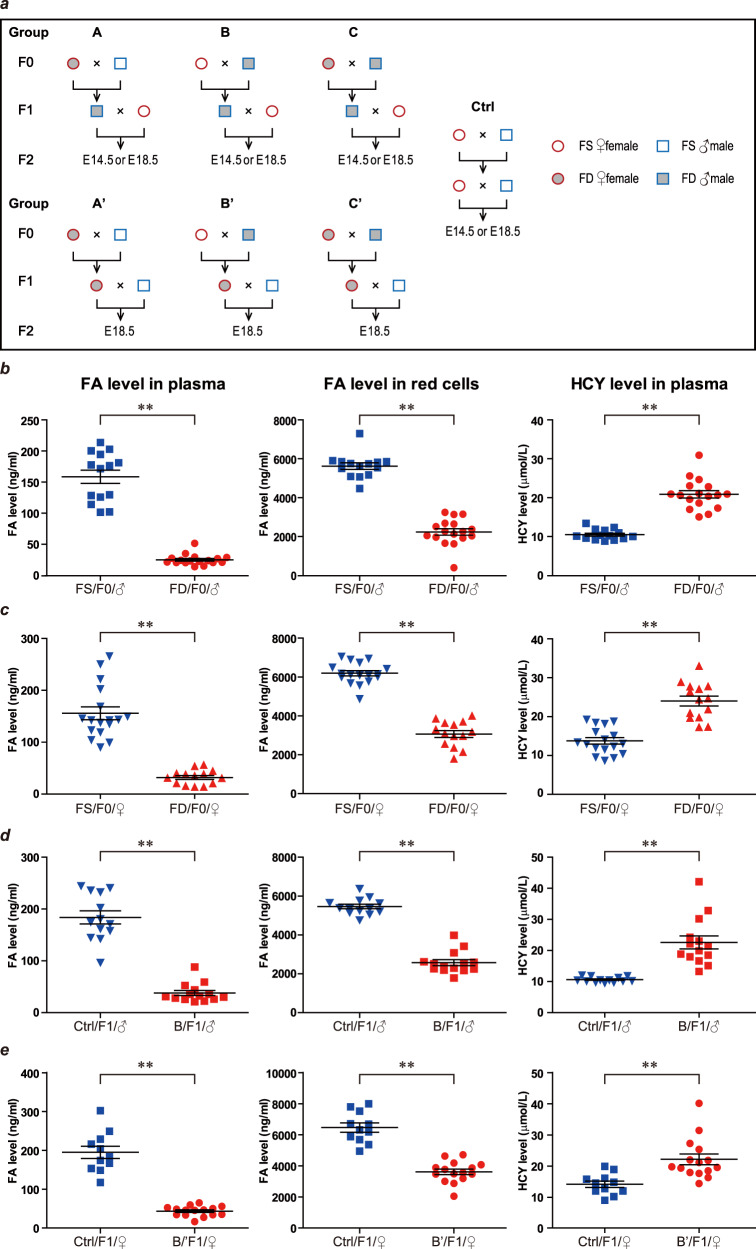
The FD diet reduced the levels of FA in plasma and red cells and increased HCY levels in plasma. **a** Brief illustration of the mice groups used in this study. Group A, B, C used F1 males fed the FD diet to cross with females fed the normal diet to produce F2; while group A’, B’, C’ used F1 females fed the FD diet to cross with males fed the normal diet to produce F2. **b**–**e** The FA levels in plasma and red cells, and HCY levels in plasma in F0 males (**b**), F0 females (**c**), F1 males in group B (**d**), and F1 females in group B’ (**e**) were examined at 20–25 weeks.

C57BL/6 mice were used in our experiments. Mice on the FD diet were put on a synthetic diet with very low FA (0.2 mg/kg) beginning at 3 weeks of age. Two continuous generations were kept on either the FD or folate-sufficient (FS) control diet that contained a normal level of FA (2.5 mg/kg) and F2 embryos were dissected and collected for further experiments. Seven groups were formed based on which parent was on the FD diet (Fig. 1a). In group A and A’, only the F0 female was on the FD diet; in group B and B’, only the F0 male was on the FD diet; and in group C and C’, both the F0 female and F0 male were on the FD diet. In groups A, B, and C, F1 males (F1♂) on the FD diet were crossed with females on the FS diet to generate F2 embryos; while in groups A’, B’, and C’, F1 females (F1♀) on the FD diet were crossed with males on the FS diet to generate F2 embryos.

**Fig. 2:**
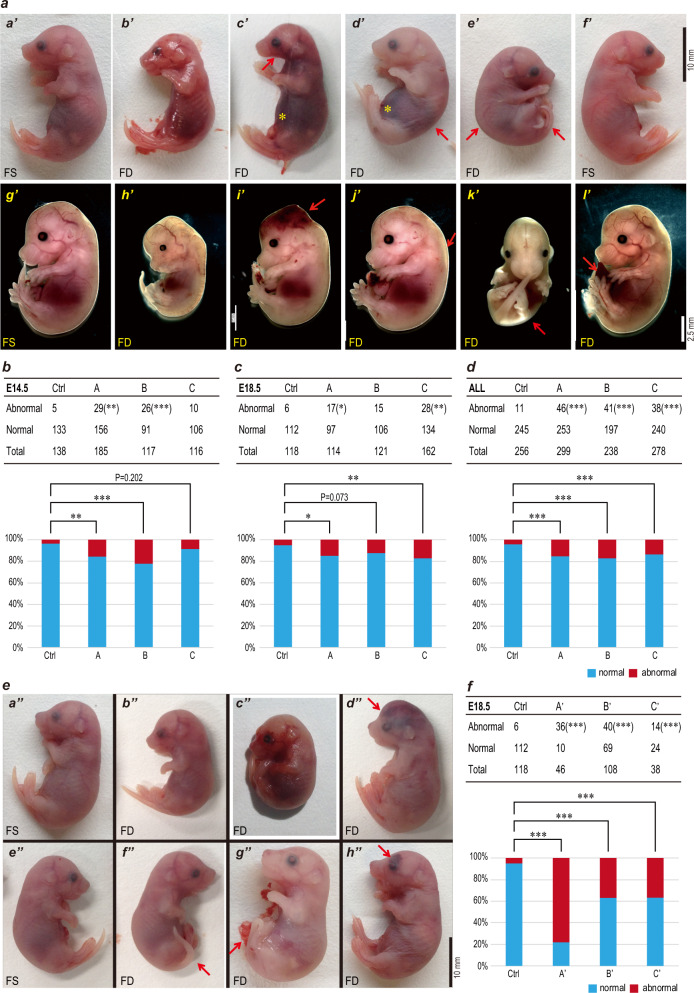
The FD diet in two generations significantly increased the malformation ratio in F2 embryos. **a** F2 embryos with typical phenotypes from group A, B, and C were shown. F2 embryos were dissected at either E18.5 (a'–f') or E14.5 (g'–l') and gross anatomy were evaluated. a', f', and g' were from control FS group and the rest embryos were from FD diet groups. Variety of defects were observed in FD diet groups, including developmental delay (b', h', k'), missing mandible (c', red arrow), curly tail (e'), scoliosis (d', e', k', red arrows), edema (j'), abdominal fissure (l'), and internal hemorrhage (yellow stars in c' and d', red arrow in i'). The amount of embryos and the abnormal ratio in either E14.5 or E18.5 were presented in panels **b** and **c**, respectively. **d** The total abnormal ratio were presented. **e** F2 embryos with typical phenotypes from group A', B', and C' were shown. Only embryos at E18.5 were dissected for these three groups. Again, variety of defects were observed in FD diet groups, including developmental delay (b'', c''), curly tail (red arrows in f'', g''), internal hemorrhage (red arrows in d'' and h'') and parietal encephalocele (d''). **f** The amount of embryos and the abnormal ratio in these three groups were presented.

To confirm the effect of the FD diet, the levels of FA and homocysteine (HCY) in plasma and FA in red cells were examined in F0 mice, F1 males in group B, and F1 females in group B’ at ~25 weeks of age. As expected, a long-term FD diet caused significant downregulation of FA and upregulation of HCY levels in blood (Fig. 1b–e). To examine the birth defects induced by FD diet, F2 embryos were collected either at E14.5 or E18.5. Gross anatomy of the embryos was examined and photos were taken for data collection. The weight of the fetus and placenta were collected and the number of corpora lutea and embryos were counted. We did not observe any difference in the number of live and total E18.5 embryos nor the ratio of total embryos to corpora lutea between the FD groups and the control group (Supplementary Fig. S1a–c), suggesting a relatively normal ovulation and fertilization process when female mice were under FD conditions. However, a variety of abnormalities were displayed in F2 embryos came from the FD diet groups, including growth retardation, internal hemorrhage, scoliosis, curly tails and so on (Fig. 2a, e). The ratios of malformed F2 embryos were significantly higher in all groups fed the FD diet than in the control group (Fig. 2b–d, f). Notably, the ratio of abnormal F2 embryos was much higher in groups with F1 females on the FD diet (group A’, B’, C’) (Fig. 2f) than in groups with F1 males on the FD diet (groups A, B, C) (Fig. 2d). A large amount of this difference came from the developmental delay and weight loss of F2 embryos when F1 females were on the FD diet (Supplementary Fig. S1d), which indirectly reflected the additional effect of FD in female mice on fetal development during pregnancy.

### WGS reveals a higher de novo mutation rate among the progeny of sibship fed the FD diet

**Fig. 3:**
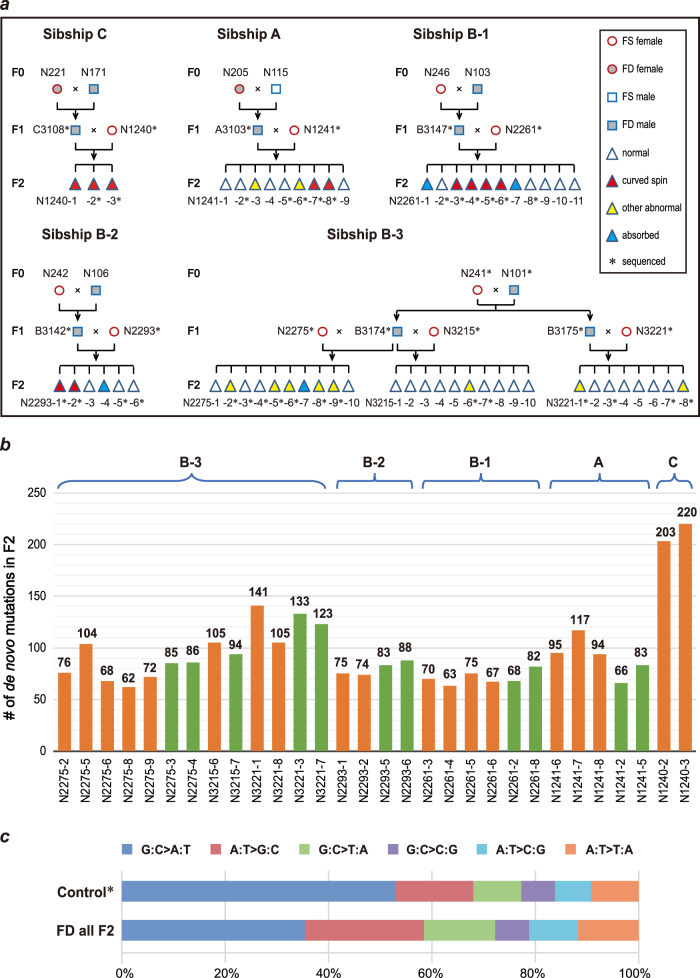
The FD diet in two generations significantly increased DNMs in F2 embryos. **a** Illustration of sibship relationships of the samples selected for whole-genome sequencing. **b** The DNMs in F2 progenies in these sibships. Samples exhibit malformations are orange, while those showing normal phenotypes are green. The average number of mutations in the A, B and C sibship are 91, 87, and 211, respectively. **c** Illustration of mutation spectra of those DNMs observed in all F2 embryos. *The control data were adopted from previous reports^22–24^.

In a previous report, higher chromosome damage and an increased mutation frequency in expanded simple tandem repeat regions were detected in sperm when male mice were fed a continuous FD diet^14^, which brings a potential risk of malformations in offspring. To measure mutations, especially DNMs, and rate changes induced by dietary FD, we collected tissue samples from 45 mice from five sibship, including one sibship from group A, three sibship from group B and one sibship from group C (Fig. 3a, sequenced samples are labeled with stars) plus 4 mice from the control group in our experiments. In sibship B-3, samples from all three generations were collected. All samples were prepared for DNA library construction and WGS with a depth of 30× for each sample. A total of 41,182 million reads (5753 Gbs) were obtained, with 356–966 million reads for each sample on average. Among them, 41,136 million reads (99.89%) were mapped onto the mouse reference genome GRCm38 (Genome Reference Consortium mouse build 38). After removal of 22.54% of the read duplications, the reads covered 95.4% of the reference genome with similar overall read distribution patterns, and yielded a total of 37,514 SNPs from 49 sequenced mice. As shown in Supplementary Fig. S2, the 49 samples were clearly classified into six groups representing sibship A, B-1, B-2, B-3, C, and the control group, on a maximum likelihood tree based on detected SNPs. These results provided cross-validation for verification of the effectiveness of the identified SNPs by comparing them with the known relationships within the sibship, and further benefited reorganization of mutations as individual, specific to or shared by associated sibship. Three generations of sequencing from the core sibship would guarantee the maximum elimination of the potential artificial positive result, which was first performed purposefully in our experimental design.

As all F1 samples in these five sibships were sequenced, we calculated the number of DNMs in F2 mice by filtering out those observed in F0 and F1 samples according to the relationship of the five sibship. The number of DNMs in F2s from groups A and B ranged from 62 to 141, with an average of 87.6 DNMs per sample (Fig. 3b). When both grandparents were on the FD diet, the number of DNMs in F2 embryos was almost doubled compared to that from one set of grandparents on the FD diet in groups A and B (Fig. 3b, group C). The reported spontaneous DNM rate in wild-type mice (C57BL/6J) per nucleotide per generation is 5.4 × 10^−^^9^, based on a 20-generation study by Uchimura et al.^22^. The mouse genome size is ~2.7E9 bp. Therefore, the expected number of spontaneous DNMs per generation per diploid is approximately 29, which is much lower than what we observed in F2 embryos. Here, our results showed that at least two times more DNMs were identified in F2 embryos when two generations were on the FD diet. We further evaluated if there is a sex bias in DNMs observed in offspring on the FD diet by comparing mutation numbers in F2 mice of sibship B1-B3. We first identified the sex of F2 embryos by comparing sequencing coverage and read depth on sex chromosomes with those on autosomes (Supplementary Table S1). Males have similar sequencing coverage and read depth on Chr. X and Y, while females have very little sequencing coverage and read depth on Chr. Y. Within the 23 F2 embryos of sibship Bs we sequenced, there are 10 male and 13 female. We then examined the mutations observed in these embryos separately (Supplementary Fig. S3). The number of DNMs in male mice ranged from 63 to 133 (median = 82.5) per mouse while that for females ranged from 62 to 94 (median = 76), suggesting that there is no significant sex bias in DNM number (*P* value = 0.33, Kolmogorov-Smirnov test).

Folate is required for the methylation process as well as for the synthesis of purines and thymidylate. Therefore, a FD diet may shift the spectra of base substitution mutations if FD causes disproportionate mutations of each type of nucleotide. The spectra of spontaneous germline base substitutions in normal C57BL/6J mice have been reported before^22–24^. The most often occurring base substitution is G:C>A:T, which is ~53% of all base substitutions. After we investigated the spectra of FD-induced substitutions in the DNMs detected in all F2 embryos, we found that the percentage of the G:C>A:T type of transition in F2 embryos of the FD sibship was ~35%, which was significantly lower than the reported 53% in mice on a normal diet^22–24^ (Fig. 3c, *P* value = 0.026, Kolmogorov-Smirnov test). Therefore, the FD diet also leads to substitution bias.

### Genotyping of DNMs inherited by F1 and F2 mice suggests that many mutations accumulate in multiple phases of reproductive development

The higher number of DNMs revealed in FD offspring leads us to trace the origin of DNMs during reproductive development. Usually, DNMs in offspring are transmitted by parents from mutated gametes, leading to germline mutations in the offspring. Alternatively, DNMs can be produced during mitosis of either zygotes or developing embryos, which were detected in mosaics. However, somatic DNMs produced during embryonic development are unlikely to be detected by WGS in this study as the average sequencing depth (~30×) across samples is not enough. The somatic DNMs can be detected in samples exhibiting deep sequencing coverage or in single-cell DNA/RNA sequencing which calls for further study. Therefore, mutations in primordial germ cells (PGCs), either homozygous or heterozygous were further investigated as follows.

**Fig. 4:**
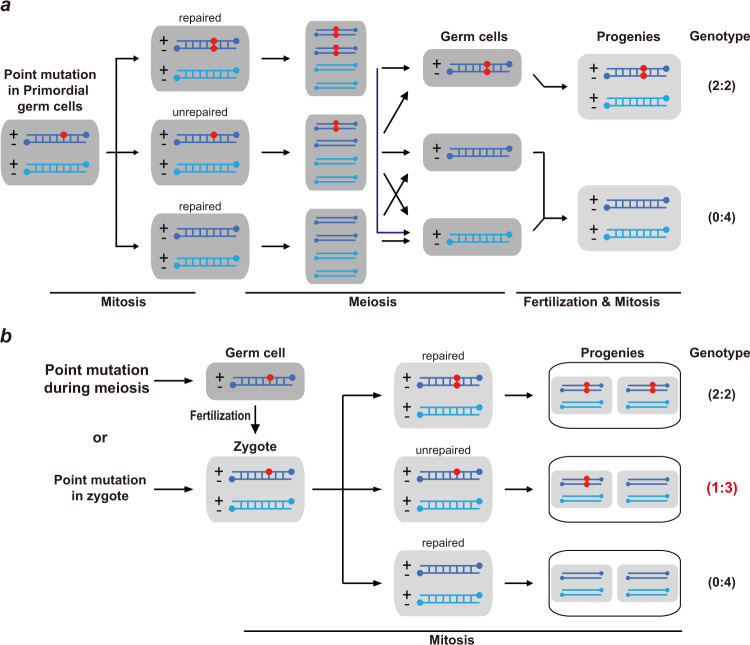
Illustration for the potential origin of DNMs based on the detected genotypes in F2 embryos. **a** When a point mutation is generated in primordial germ cells, whether it is repaired or not during mitosis and meiosis, the locus of a progeny must has a genotype as either 2:2 or 0:4. **b** When a point mutation is accumulated during meiosis or in zygote, the locus of a progeny has a genotype as 2:2 (or 0:4) when this mutation is repaired. When this mutation is not repaired, the locus of a progeny has a genotype as 1:3.

**Fig. 5:**
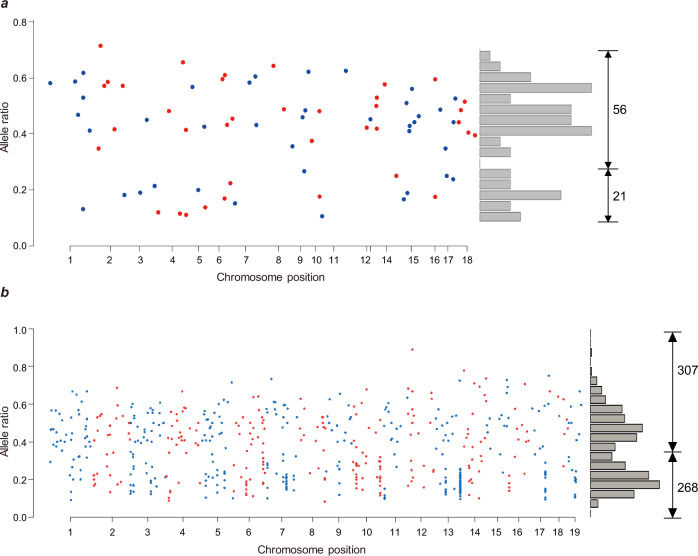
The allele ratio of DNMs detected in F1s and F2s of the B-3 sibship. **a** DNMs of F1 mice are ordered by their positions on genome, with alternative colors for different chromosomes. Among them, 56 SNVs associate with allele ratio close to 0.5 (2:2), the other 21 SNVs with ratio close to 0.25 (1:3). **b** DNMs observed in F2 embryos, among which 307 SNVs associate with an allele ratio close to 0.5 (2:2), the other 268 SNVs with ratio close to 0.25 (1:3).

**Fig. 6:**
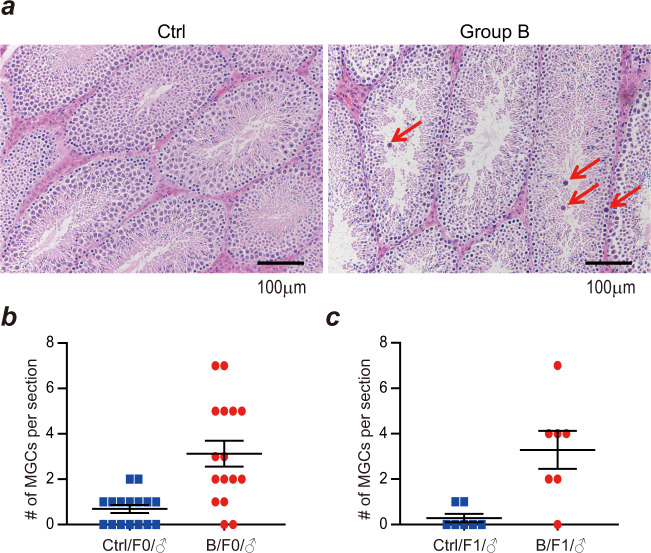
IHC of testis from control male and group B male testes. **a** Tipic IHC on testes sections from two groups were shown here. The multinucleated giant cells (MGCs) were pointed out by red arrows. The scale bar is 100 μm. **b**, **c** The number of MGCs per section were counted and the average number is significantly high in group B than that in control. The number in F0 males (**b**) and in F1 males (**c**).

PGCs undergo multiple rounds of cell division to generate mature germ cells. A novel substitution obtained in a PGC leads to a change in nucleotide in one of two DNA strands. The nucleotide mismatch between two strands can be resolved during mitosis by using the mutated strand as a model strand, and the mutation is carried by both strands in divided cells. Consequently, the mutation is homozygous in germ cells after genome replication in meiosis and shows an allelic ratio of 2:2 in progeny genomes, of which both DNA strands are inherited from the mutated parent. Alternatively, the mutation is fixed during mitosis when using the unaffected strands as template strands, leaving no trace in the genomes of either germ cells or zygotes. Otherwise, the mismatch can be ignored for DNA repair during mitosis and is retained until genome replication in meiosis. In summary, a mutation accumulated before the formation of germ cells displays a phenotype of 2:2 (i.e., allelic ratio of 50%) or 0:4 (i.e., no observed mutation) in sequencing data of progeny genomes (Fig. 4a). As in the large sibship B-3, we sequenced all three generations (Fig. 3a, Supplementary Fig. S4a), and we detected DNMs in both F1 and F2 individuals by ignoring the mutations identified in F0 mice. A total of 77 DNMs were detected in F1 males B3174 and B3175, and 575 DNMs were detected in F2 mice in sibship B-3 (Supplementary Fig. S4b). Among the 77 DNMs detected in the two F1 males of sibship B-3, 56 of them (72.7%) were associated with an allelic ratio of 50% (range from 30% to 70%) (Fig. 5a). However, the other 21 DNMs have allelic ratios of 10–30%, and these are likely to have a genotype at 1:3, namely, only one of four DNA strands would have a remaining novel mutation (Fig. 5a). Similar results were also observed in the DNMs in F2, as 307 out of 575 DNMs had genotype close to 1:1, and the other 268 DNMs had a genotype close to 1:3 (Fig. 5b). Therefore, we offer an alternative model for this type of mutation accumulation (Fig. 4b). Novel mutations might occur in one of two strands in germ cells or zygotes, yielding DNA mismatches as described above. A mismatch ignored by DNA repair mechanisms is unresolved until next round of cell division during mitosis, to segregate the heterozygous base pair through genome replication. Therefore, half of diploid cells exhibit the mutation on one allele as homozygous (showing a genotype of 1:1), while the other cells have no mutation (as a genotype of 0:2). Taken together, the results of high-throughput sequencing of the mixture of all embryonic cells showed a phenotype of 1:3 (Fig. 4b). These results indicate defectiveness of DNA repair mechanisms when undergoing continuous FD, resulting in mutations accumulating during the formation of germ cells (meiosis) and zygotes. As defectiveness in DNA repair during meiosis could also lead to arrested spermatogenesis and the formation of multinucleated giant cells in the testis^25,26^, we performed sectioning and HE staining on the testes from the FD and control groups. As shown in Fig. 6, the number of multinucleated giant cells in FD males was significantly greater than that in control males, although there was no change in the weight of the testis and epididymis (data not shown).

### Potential hotspots of DNMs relating to folate stress

Novel mutations can be distributed unevenly along the chromosomes, leading to potential hotspots of mutations in short regions. All the mutations of all F2 samples are summarized for counting the distribution of mutations on 19 mouse chromosomes (Supplementary Fig. S5a), revealing that 65 windows (10 Mb) are associated with 14 or more mutations. We found that the distribution did not agree with a Poisson model (Supplementary Fig. S5b), indicating the presence of hotspots of novel mutations. As genetic studies suggested that double-strand breaks (DSBs), a key intermediates of mitotic or meiotic recombination, might be unevenly distributed along chromosomes, the positions of those novel mutations yielded by falsely repaired DSBs might be affected. Nevertheless, we found a total of 34 genes associated with nonsynonymous substitutions caused by novel mutations in exon regions in the FD group (Supplementary Table S2). Although these genes showed no GO function enrichment (Supplementary Table S3), consistent with diverse developmental defects observed among F2 mice, many of them were reported to be essential for normal development, e.g., the gene *Ptk7* coding for protein tyrosine kinase was associated with NTDs^27,28^. It is worth noting that three of those 34 genes are novel genes with unknown function. The homologs of these three novel genes commonly presented in Muroidea, vertebrate, or chordates^29^, respectively (Supplementary Fig. S6). The novel gene Gm5724 belong to gene family which is originated from a common ancestor of chordates and has subsequently undergone multiple rounds of gene duplication events. The presence of multiple homologous of Gm5724 in the mouse genome after the divergence of rodent and primate suggests both functional redundancy and divergence of the gene family^30^.

As for sibship B-3, only one of the 77 DNMs in F1 mice locates in exonic region of a gene *Creg1*, supporting the finding that F1 mice lack obvious abnormal phenotype. In comparison, exonic regions of ten genes are affected by DNMs in F2 mice (Supplementary Fig. S7), including one deletion and three nonsynonymous mutations (deleterious mutations with PROVEAN score < −2)^31^. We used the STRING database to predict the interaction of the proteins encoded by these ten genes^32^. Among them, Ptk7 and Gnai1 are involved in complex interaction networks, while another two (Hmcn2 and Exd1) are central nodes of star-like networks, suggesting mutations on these genes are unlikely to be tolerant than the others with less links, e.g., the Creg1 which was affected in F1 mice, interacting with only one protein (Supplementary Fig. S8).

### More SVs detected in FD samples further show decreased DNA repair efficiency

Large-scale structural variants (SVs), including insertions, deletions, and copy number variants (CNVs), increase genome complexities between individuals and are often involved in genetic disorders, including developmental delays and intellectual disabilities. To compare the number of SVs in sampled mice affected by FD with those of samples as a control, large deletions and CNVs were detected according to sequencing depth and paired-end information of sequencing data from sibship B-3, which contains three generations. As shown in Fig. S9A, among the 2,121 deletions found in sibship B-3, 42 to 195 deletions were found in F2 samples with developmental defects (N2275-2/-5/-6/-8/-9) compared with four samples in the F0 and F1 generations, while only a few (11 to 22) deletions were found in the other two siblings which appeared normal (N2275-3/-4). Most of these deletions ranged from 50 bp to 200 bp (Supplementary Fig. S10a), being shorter than the length of meiotic DSBs (300 bp to 1.8 kb), suggesting that DSB-oriented deletions are partially repaired after the resolution of meiotic recombination or other possibilities. Furthermore, we detected 39 to 108 CNVs with lower sequencing coverage in F2 mice with defects (N2275-2/-5/-6/-8/-9) compared with four samples in the F0 and F1 generations, which was also more than the 15–32 CNVs in other F2 mice (N2275-3/-4) (Supplementary Fig. S9b). The duplications ranged from 100 bp to 1000 bp (Supplementary Fig. S10b). It is worth noting that transposable elements (TEs) are indeed affected by the majority of deletions and CNVs though differed in numbers and sizes in different TE categories. (Supplementary Fig. S9c, d). The finding of more deletions and a possible increased copy number among CNVs also suggested reduced DNA repair function, which calls for further studies.

## Discussion

The amount of folate in the diet has a profound impact on reproductive outcomes, and a FD diet can cause negative reproductive outcomes. Previous studies have shown that the number of DNMs is associated with negative reproductive outcomes, including many developmental disorders in offspring. Nevertheless, it remains elusive whether a FD diet causes DNMs and whether this contributes to negative reproductive outcomes. The rapid development of sequencing techniques provides us with the opportunity to investigate this matter. Here, we used C57BL/6 mice to study the effect of a FD diet on DNMs in F2 individuals produced by F1 males receiving FD diets considering that the majority of DNMs arise in the paternal lineage^18–20^ and that F2 embryos came from F1 females on FD diets have severe weight loss/developmental delay due to nutritional restriction. The reported spontaneous DNMs in wild-type mice under normal conditions is ~29–36 per generation per diploid, which has been tested in different groups with different strains of mouse^22,33,34^. However, we found that ~90 DNMs in F2 embryos came from one F0 sex and F1 male on FD diets regardless of whether they appeared normal or abnormal. Moreover, the number of DNMs in F2 embryos doubled when both grandparents were fed the FD diet (~200 versus ~90) (Fig. 3b). The number of DNMs in our experiments was ~3–7 times of that in mice fed a normal diet. Most importantly, the identified number of DNMs in our experiment was based on familial analysis of sequencing data of three continuous generations (F0, F1, and F2), which guaranteed that we would eliminate false calling results with extreme confidence levels. What will the number of DNMs be when a similar situation happens in humans? Considering that the reported spontaneous mutation rate in humans is 1.2 × 10^−8^ per nucleotide per generation^18,22^ and the human genome size is 3.2 × 10^9^ nt, the expected DNMs in humans would be approximately 70 nt per generation per diploid. If the same ratio (3–7 times) was to occur in humans on a FD diet, it would yield approximately 210 to 490 DNMs per diploid per generation. Furthermore, it was estimated that there are 2.1 deleterious mutations within 70 DNMs per diploid^18,22^, which would result in 6–15 deleterious DNMs in humans on a FD diet. As more deleterious mutations are associated with a higher risk for birth defects^35^, this increased number of DNMs induced by FD diet would increase the risk for birth defects. Therefore, it was speculated that a FD diet in humans might also increase the number of DNMs in sperm, which helps to explain FD-induced negative reproductive outcomes.

An interesting observation in our study was many DNMs detected in F2 individuals exhibiting a 1:3 genotype. This genotype ratio of DNMs is likely due to mutations generated during spermatogenesis remaining unrepaired during early zygote development (Fig. 4b). Therefore, our study indicated that sufficient folate is essential to maintain a normal DNA repair function during the entire reproductive life of men and during early pregnancy in women. The FD diet can cause defects in DNA repair, which is further supported by the increased CNVs in F2 embryos and demonstrated by the multinucleated cells in the testes of FD males.

We also revealed a shift in the DNM spectra detected in the F2 generation, namely, the most commonly observed transition, G:C to A:T, was much less common in FD mice (35.4%) than in C57BL/6J mice fed a normal diet, as reported previously (53%)^23^. These G:C>A:T transitions are normally the most frequent mutations, and one of the reasons for such high frequency is due to the high mutability of CpG dinucleotides^23,36^. The cytosine in the CpG site is often methylated to form 5-methylcytosine, which undergoes deamination as a result of spontaneous oxidation^18,23^. It has been suggested that higher methylation levels are associated with higher mutation rates of CpG dinucleotides during germline development^37^. Therefore, a FD diet could lead to a globally lower methylation level and fewer genome-wide G:C>A:T transitions. Additionally, the FD diet could cause defects in DNA repair by reducing purines and dTTP in cells. These mismatches are efficiently repaired by a base excision repair mechanism or, alternatively, retained as mutations in genomes due to reduced DNA repair ability under a FD diet.

Here, we demonstrated that the FD diet had a severe impact on reproductive outcomes and increased the amount of DNMs during spermatogenesis. The FD diet has such adverse effects, likely due to not only the decreased level of methylation but also defective DNA repair. Although the effect of excessive folate in the diet was not assessed in this study, concerns have recently been raised about possible adverse effects of high folate intake. It has been shown that a 10-fold higher FA supplementation in female mice can cause many fetal developmental delays and defects^38^. Another recent study also showed that not only FD but also excess folate in female mice can lead to delayed prenatal cerebral cortical neurogenesis due to disruptions in folate metabolism of the offspring^39^. Taken together, these data indicate that it is of essential importance to ensure the intake an appropriate amount of folate for both sexes during their reproductive years. As much knowledge has been obtained regarding the advantages of maternal FA supplementation to reduce birth defects, more should be learned on the regulation of FA balance from the paternal side as a potential effective measure to further reduce the total incidence of birth defects.

## Material and methods

### Animal and sample collection

Mice were maintained in the animal facility at Sino-British SIPPR/B&K Lab Animal Ltd. The protocol was approved by the Committee on the Ethics of Animal Experiments of Sino-British SIPPR/B&K Lab Animal Ltd. (Ethics approval number: 2016013). The FD diet was obtained from Shanghai Pulutan Biotechnology Co., LTD (China). The FD diet contained 0.2 mg/kg FA and the control folate sufficient (FS) diet contained 2.5 mg/kg FA. Both diets contained approximate 20% protein, 66% carbohydrate (CHO), and 5% fat by weight. The formula feeds were prepared following the PRC National standard #GB14924.3-2010 “Nutrients for formula feeds for laboratory animals”. C57BL/6 mice were put on the FD diet beginning at 3 weeks of age. F2 embryos were dissected at E14.5 or E18.5. Gross anatomy of the embryos were examined and photos were taken for data collection. The weight of the fetus and placenta were collected and the number of corpora lutea were counted. After gross anatomy, the tail and one foot of each fetus were collected for genomic DNA extraction. The rest of the embryos were collected separately and stored at −80 °C. Testes were collected from F0 and F1 males on the FD or FS diet and subjected to routine HE staining.

### Folate and HCY detection

Blood was collected from mice at 20–25 weeks of age. Approximately 500 μl of blood was collected from each mouse in an anticoagulated tube. After 30 min at room temperature, whole blood was centrifuged at 2500 *g* for 10 min. A total of 250 μl plasma of each sample was collected and transferred to a new tube. The level of HCY in whole blood was detected by the Sheath-flow resistance method on a hematology analyzer, XT-2000i, Sysmex. The levels of FA in both whole blood and plasma were detected by chemiluminescence immunoassay using a Folic Acid test kit on an automatic chemiluminescence immunoanalyzer (IMMULITE 2000 XPi, Siemens). The levels of FA in red blood cells were calculated by means of the following formula: FA red blood cell = FA whole blood/HCY*100-(FA plasma*(100-HCY)/HCY). The levels of HCY in plasma were detected by enzymatic cycling assay using the HCY test kit on a biochemical analyzer (7600, HITACHI).

### Whole genome sequencing (WGS)

Genomic DNA from each test subject was isolated from tissue samples using conventional reagents and was quantified using a NanoDrop2000 (Thermo Scientific). The whole genome was sequenced using the Illumina ×10 platform according to the manufacturer’s instructions by Beijing Genomics Institution.

### Mapping of NGS short reads and quality control

The mouse reference genome (UCSC version mm10) and corresponding gene annotations were downloaded from the UCSC webserver. Sequenced reads for 51 individuals were trimmed by Trimmomatic and FastQC, and high-quality reads were mapped against the reference by BWA (version 0.7.10-r789)^40,41^ using the MEM model and default parameters. Reads with mapping quality scores >20 were considered uniquely mapped and were further filtered out by SAMBLASTER (version 0.1.22)^42^ to remove PCR duplicates. Discordantly mapped read pairs were kept for further analyzes.

### Primary identification of single nucleotide variants from samples

Raw SNVs were detected by using FreeBayes (version v0.9.21)^43^ with options of “–min-repeat-entropy 1” for each sample. A qualified SNP must pass the following filtering procedures: 1) being supported by a proper sequencing depth (20–60×), within three standard deviations of the mean coverage; 2) mutant alleles that are supported by three or more short reads; 3) a minimum quality of 30 for each SNV candidate; 4) a mean mapping quality of both alleles on a given locus that exceeds 30; and 5) use of Tandem Repeats Finder (version 4.09)^44^ to detect tandem repetition of nucleotides with options of “2 7 7 80 10 50 500”, with SNVs overlapping with tandem repeat regions being removed by inGAP-sv.

### Further filtering of SNVs based on trio relationships of samples

Not all artificial SNPs were excluded from the above procedure; in addition, natural mutations that accumulated early and were shared by all mouse sibship involved in this study needed to be removed. Here, we present a brief description of the basic procedures for the prediction and inspection of SNVs exhibited during the stages of F0 to F1 to F2. The primary predicted SNVs of all sibship were collected to build a supermatrix, and a phylogenetic tree of all samples was reconstructed by using the maximal likelihood method in IQTree (version 1.6.12)^45^ with a model of “GTR + F + R4”, which is the best-fit model detected by ModelFinder^46^. Those SNVs on the root of the tree, namely, those that were highly likely to be shared by all samples, including four samples without the underlying stress of FD, were removed from further analyzes. Third, novel SNVs exhibited by F2 progenies were recognized by comparing allelic ratios in parental genomes: F0 samples (for sibship B) and F1 samples (for all sibship). Finally, all predicted SNVs were examined manually to remove artificial SNVs due to SVs and other genome complexities. SNVs that passed the filter were annotated by ANNOVAR (version 20170717)^47^ for each sample.

### SV detection and functional analyses

inGAP-sv^48^ and CNVnator (version v0.3.3)^49^ were employed to identify SVs, including deletions and CNVs larger than 100 bp, based on mapping information of paired-end reads, split reads and coverage depth. Predicted SVs are evaluated to reduce false-positive predictions as follows: mapping of short reads was recounted by BEDTools (version 2.26.0)^50^ for measurement of sequencing coverage on predicted SV regions; detected SVs adjacent to centromere or telomere regions are ignored; SVs exhibit read coverage 50% lower or higher than both average values and that of the flanking region was kept for further analyzes. Average sequencing depth are normalized for each sample of a sibship to calibrate estimated read depth of each SV candidate. SVs exhibiting 0.5× or higher difference on read depth between a F2 genome and one of its parental genome are colored in red in Supplementary Fig. S9.

## Supplementary information

Supplementary materials
