# Supplementary material for: Parental folate deficiency induces birth defects in mice accompanied with increased de novo mutations
Source: Cell Discov. 2022 Feb 22;8:18. doi: 10.1038/s41421-021-00364-0 (PMC8861018; doi:10.1038/s41421-021-00364-0)
Supplement: Supplementary file 1 — Supplementary materials [file 41421_2021_364_MOESM1_ESM.pdf]

Figure legends

Figure 1. The FD diet reduced the levels of FA in plasma and red cells and increased HCY levels in plasma. (**a**) Brief illustration of the mice groups used in this study. Group A, B, C used F1 males fed the FD diet to cross with females fed the normal diet to produce F2; while group A', B', C' used F1 females fed the FD diet to cross with males fed the normal diet to produce F2. The FA levels in plasma and red cells, and HCY levels in plasma in F0 males (**b**), F0 females (**c**), F1 males in group B (**d**), and F1 females in group B' (**e**) were examined at 20-25 weeks.

Figure 2. The FD diet in two generations significantly increased the malformation ratio in F2 embryos. F2 embryos with typical phenotypes from group A, B, and C were shown in (**a**). F2 embryos were dissected at either E18.5 (a'-f') or E14.5 (g'-l') and gross anatomy were evaluated. a', f', and g' were from control FS group and the rest embryos were from FD diet groups. Variety of defects were observed in FD diet groups, including developmental delay (b', h', k'), missing mandible (c', red arrow), curly tail (e'), scoliosis (d', e', k', red arrows), edema (j'), abdominal fissure (l'), and internal hemorrhage (yellow stars in c' and d', red arrow in i'). The amount of embryos and the abnormal ratio in either E14.5 or E18.5 were presented in (**b**) and (**c**) respectively. The total abnormal ratio were presented in (**d**). F2 embryos with typical phenotypes from group A', B', and C' were shown in (**e**). Only embryos at E18.5 were dissected for these three groups. Again, variety of defects were observed in FD diet groups, including developmental delay (b'', c''), curly tail (red arrows in f'', g''), internal hemorrhage (red arrows in d'' and h'') and parietal

encephalocele (d”). The amount of embryos and the abnormal ratio in these three groups were presented in (**f**).

Figure3. The FD diet in two generations significantly increased DNMs in F2 embryos. (**a**) Illustration of sibship relationships of the samples selected for whole genome sequencing. The DNMs in F2 progenies in these sibship were presented in (**b**). Samples exhibits malformations are orange, while those showing normal phenotypes are green. The average number of mutations in the A, B and C sibship are 91, 87 and 211, respectively. (**c**) Illustration of mutation spectra of those DNMs observed in all F2 embryos. *The control data were adopted from previous reports [22-24].

Figure 4. Illustration for the potential origin of DNMs based on the detected genotypes in F2 embryos. (**a**) When a point mutation is generated in primordial germ cells, whether it is repaired or not during mitosis and meiosis, the locus of a progeny must has a genotype as either 2:2 or 0:4. (**b**) When a point mutation is accumulated during meiosis or in zygote, the locus of a progeny has a genotype as 2:2 (or 0:4) when this mutation is repaired. When this mutation is not repaired, the locus of a progeny has a genotype as 1:3.

Figure 5. The allele ratio of DNMs detected in F1s and F2s of the B-3 sibship. (**a**) DNMs of F1 mice are ordered by their positions on genome, with alternative colors for different chromosomes. Among them, 56 SNVs associate with allele ratio close to 0.5 (2:2), the other 21 SNVs with ratio close to 0.25 (1:3). (**b**) DNMs observed in F2 embryos, among which 307 SNVs associate with an allele ratio close to 0.5 (2:2), the other 268

SNVs with ratio close to 0.25 (1:3).

Figure 6. IHC of testis from control male and group B male testes. (a) Tipic IHC on testes sections from two groups were shown here. The multinucleated giant cells (MGCs) were pointed out by red arrows. The scale bar is 100μm. (**b-c**) The number of MGCs per section were counted and the average number is significantly high in group B than that in control. (**b**) is the number in F0 males and (**c**) is in F1 males.

Supplementary Figure S1. The number of live mice and total mice, the ratio of total embryos to corpora lutea, and the weight of E18.5 mice were counted for all seven groups.

Supplementary Figure S2. A maximal likelihood tree of 49 samples constructed based on predicted SNVs. Individuals of the same sibship (colored circles) are well clustered without any outlier.

Supplementary Figure S3. The number of DNMs observed in male and female F2 mice of sibship B1-3.

Supplementary Figure S4. The sibship tree of B-3 (**a**) and the number of DNMs in F1 males and filtered DNMs in F2 embryos (**b**). Samples exhibits malformations are orange, while those showing normal phenotypes are green.

Supplementary Figure S5. (**a**) Distribution of SNVs detected in all F2s are shown along chromosomes by sliding windows with size of 10 Mb and step of 5 Mb. (**b**) A histogram

presents the number of samples exhibiting SNVs per window in (A). The orange bins are the potential mutation hotspots.

Supplementary Figure S6. Illustration of phylogenetic relationship of three functional unknown genes with their homologs in animals. (**a**) Gm5724; (**b**) Gm35315; and (**c**) Gm3667 with homologs presented on the left and annotated domains presented on the right side. Homologs of the three genes are provided by Ensembl GeneTree database. Domains are annotated by using hmmseach compared with sequences in Pfam database.

Supplementary Figure S7. Distribution of DNMs detected in F2 progenies of the B-3 sibship along chromosomes. The DNMs in F2 embryos on the genome were labeled on cycles (with alternating yellow and gray backgrounds), each of which represents a sample within one sub-sibship. The number of three sub-sibship of B-3 was labeled on three inner cycles. Red bars on each cycle denote mutations found in F1 mice and were further retained in F2 mice, green bars denote DNMs found in F2 mice. Gene labeled outside are those with nonsynonymous mutations in F2 embryos of the B-3 sibship.

Supplementary Figure S8. Illustration of protein interactions of genes affected by DNMs detected in F1 and F2 mice of B-3 sibship. Protein interaction information are provided by the STRING database. Red nodes denote for protein encoded by genes with DNMs.

Supplementary Figure S9. Illustrations of CNVs detected in sibship B-3. The deletions (**a**) and duplications (**b**) detected in sibship B-3 compared with the mouse reference

genome are shown as dot-plots. The normalized read coverage of deletions and duplications in selected F2 embryos (N2275-3, N2275-4, N2275-2, N2275-5, N2275-6, N2275-8, N2275-9) were compared with two F0 mice (N101 and N241) and two F1 mice (B3174 and N2275). Red dots denote for deletions/duplications exhibiting 0.5X or more changes on read depth between paired samples. Display of transposable elements of each category affected by deletions (**c**) and CNVs (**d**), respectively.

Supplementary Figure S10. Illustration of the length distribution of the deletions (**a**) and duplications (**b**) detected in sibship B-3.

Supplementary Table S1. The coverage and read depth on each chromosomes for all 45 sequenced samples within sibship A-C.

Supplementary Table S2. The list of 34 genes associated with nonsynonymous substitutions caused by DNMs in exon regions in the FD mice.

Supplementary Table S3. GO function analysis for the 34 genes associated with nonsynonymous substitutions caused by DNMs.

Figure S1

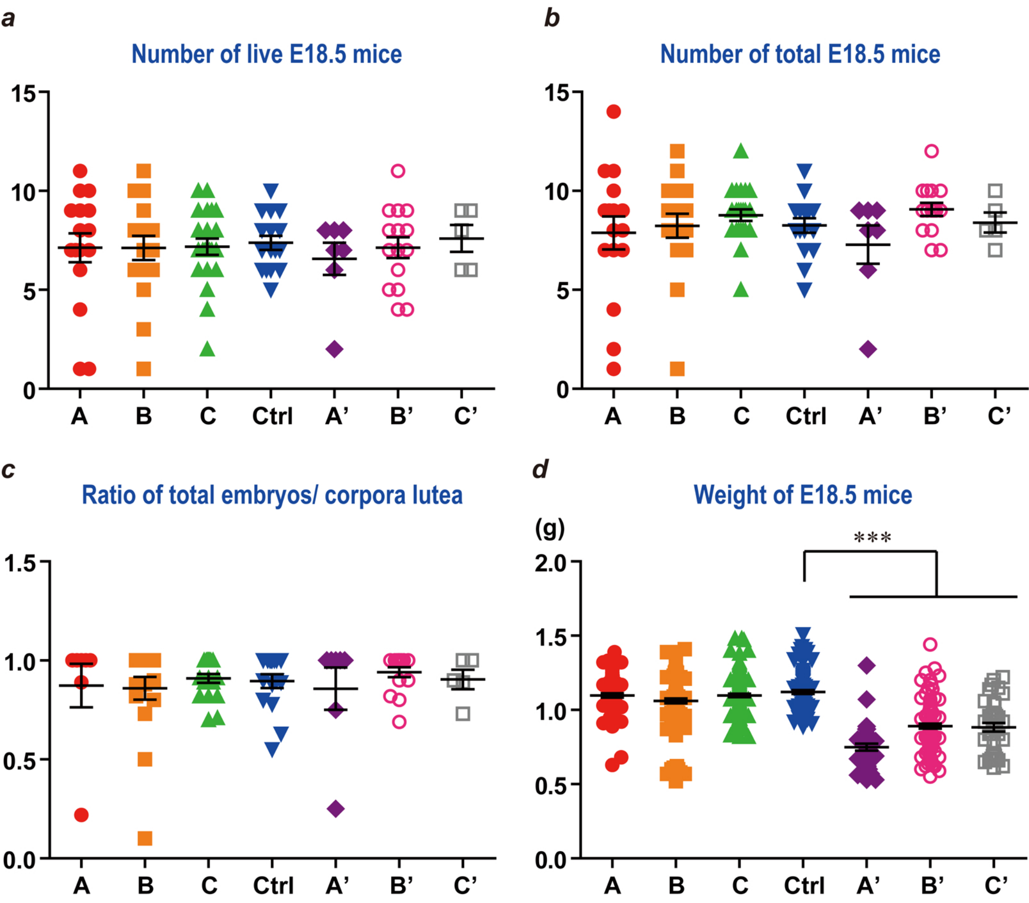
a
Number of live E18.5 mice
15
10
5
0
A
B
C
Ctrl
A'
B'
C'
b
Number of total E18.5 mice
15
10
5
0
A
B
C
Ctrl
A'
B'
C'
c
Ratio of total embryos/ corpora lutea
1.5
1.0
0.5
0.0
A
B
C
Ctrl
A'
B'
C'
d
Weight of E18.5 mice
(g)
***
2.0
1.5
1.0
0.5
0.0
A
B
C
Ctrl
A'
B'
C'

Figure S2

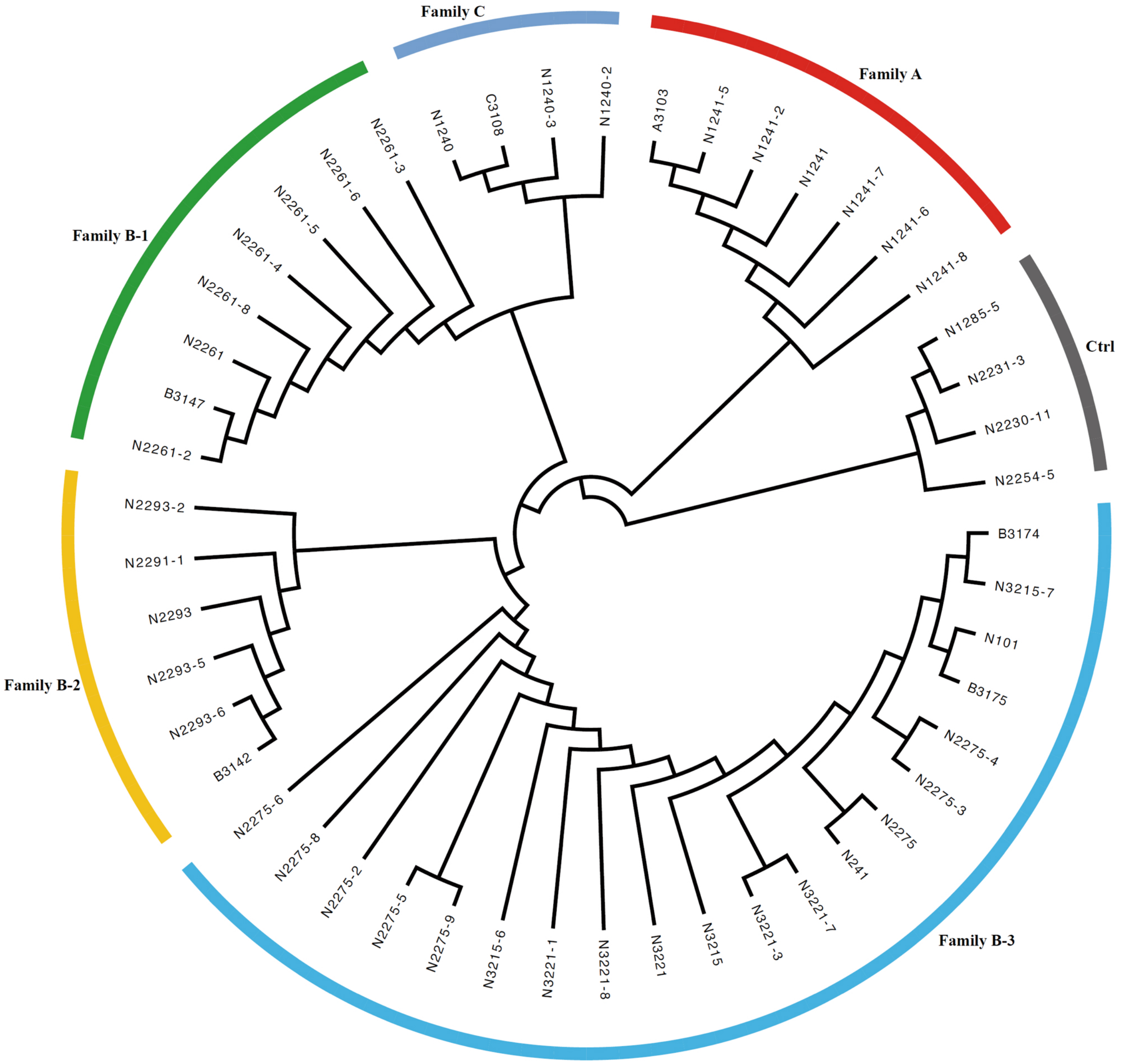
Family C
Family A
Family B-1
Ctrl
Family B-2
Family B-3
N1240
C3108
N1240-3
N1240-2
A3103
N1241-5
N1241-2
N1241
N1241-7
N1241-6
N1241-8
N1285-5
N2231-3
N2230-11
N2254-5
N2261-3
N2261-6
N2261-5
N2261-4
N2261-8
N2261
B3147
N2261-2
N2293-2
N2291-1
N2293
N2293-5
N2293-6
B3142
B3174
N3215-7
N101
B3175
N2275-4
N2275-3
N2275
N241
N3221-7
N3221-3
N3215
N3221
N3221-8
N3221-1
N3215-6
N2275-9
N2275-5
N2275-2
N2275-8
N2275-6

Figure S3

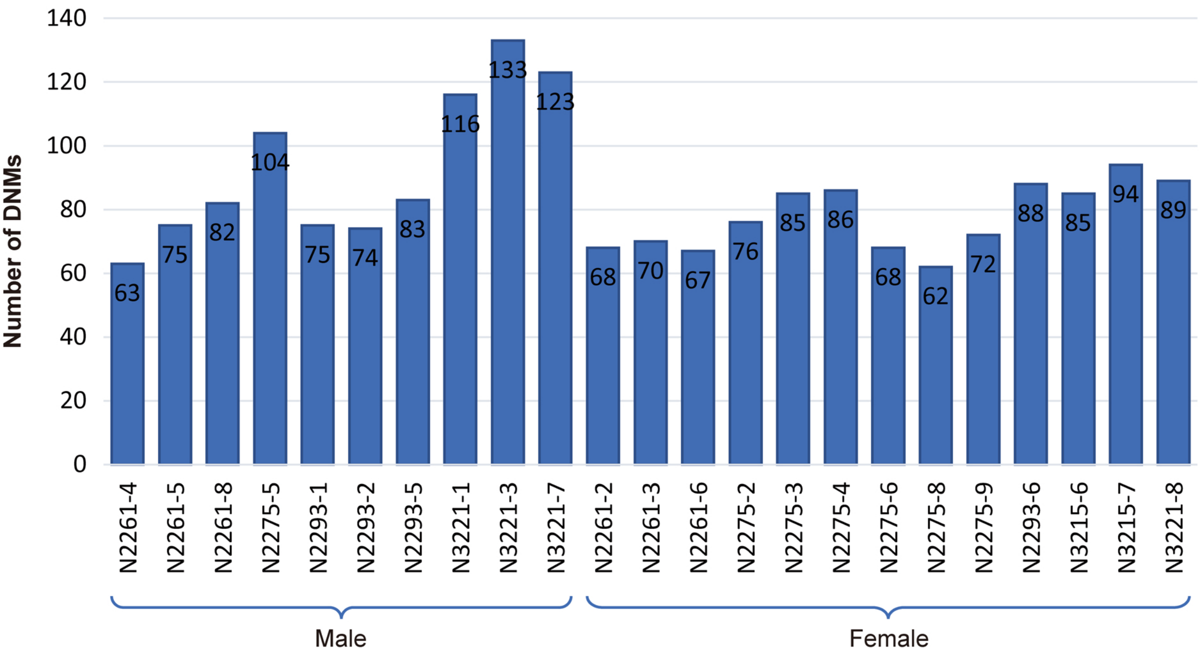

140
120
100
80
60
40
20
0
Number of DNMs
63
75
82
104
75
74
83
116
133
123
68
70
67
76
85
86
68
62
72
88
85
94
89
N2261-4
N2261-5
N2261-8
N2275-5
N2293-1
N2293-2
N2293-5
N3221-1
N3221-3
N3221-7
N2261-2
N2261-3
N2261-6
N2275-2
N2275-3
N2275-4
N2275-6
N2275-8
N2275-9
N2293-6
N3215-6
N3215-7
N3221-8
Male
Female

Figure S4

a

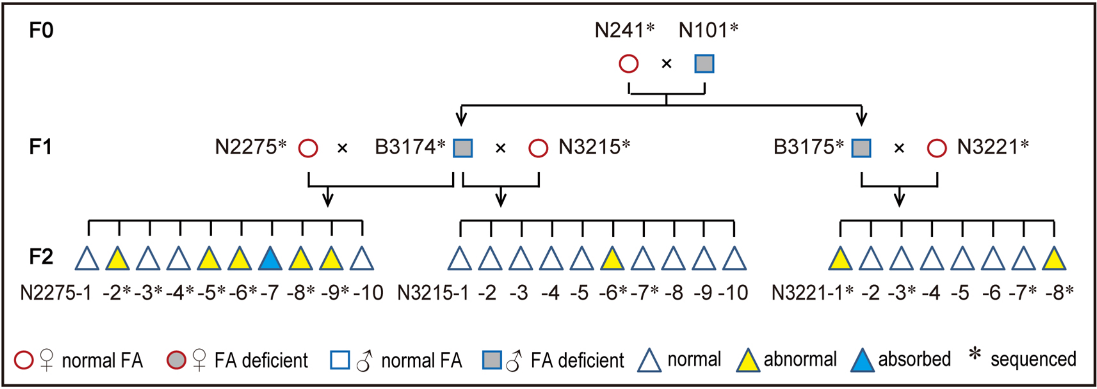
F0
N241* N101*
F1
N2275* B3174* N3215*
B3175* N3221*
F2
N2275-1 -2* -3* -4* -5* -6* -7 -8* -9* -10
N3215-1 -2 -3 -4 -5 -6* -7* -8 -9 -10
N3221-1* -2 -3* -4 -5 -6 -7* -8*
♀ normal FA
♀ FA deficient
♂ normal FA
♂ FA deficient
normal
abnormal
absorbed
* sequenced

b

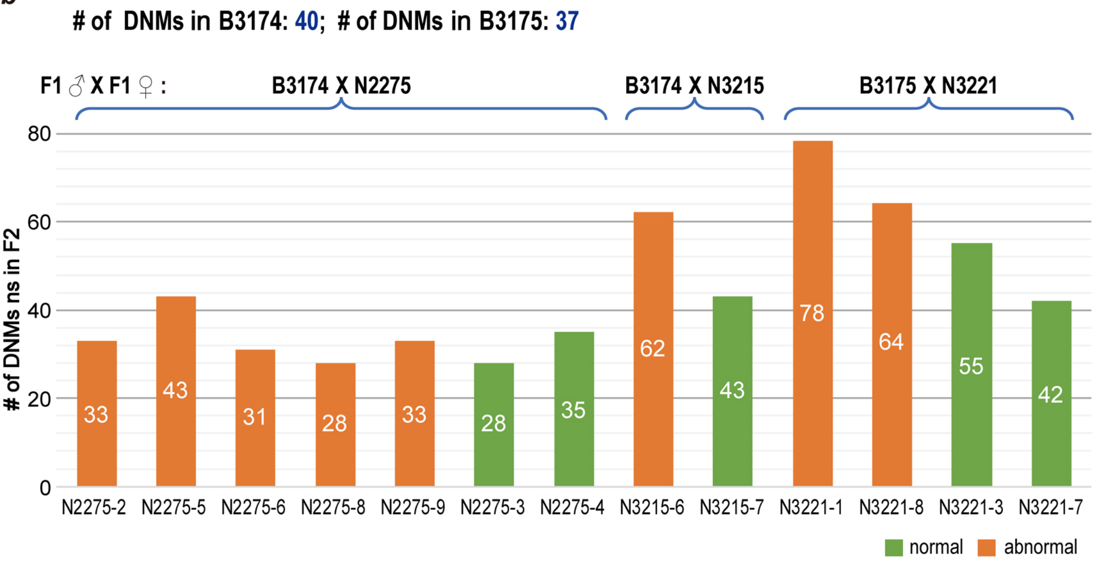
# of DNMs in B3174: 40; # of DNMs in B3175: 37
F1 ♂ X F1 ♀ :
B3174 X N2275
B3174 X N3215
B3175 X N3221
# of DNMs ns in F2
80
60
40
20
0
33
43
31
28
33
28
35
62
43
78
64
55
42
N2275-2
N2275-5
N2275-6
N2275-8
N2275-9
N2275-3
N2275-4
N3215-6
N3215-7
N3221-1
N3221-8
N3221-3
N3221-7
normal
abnormal

Figure S5

a

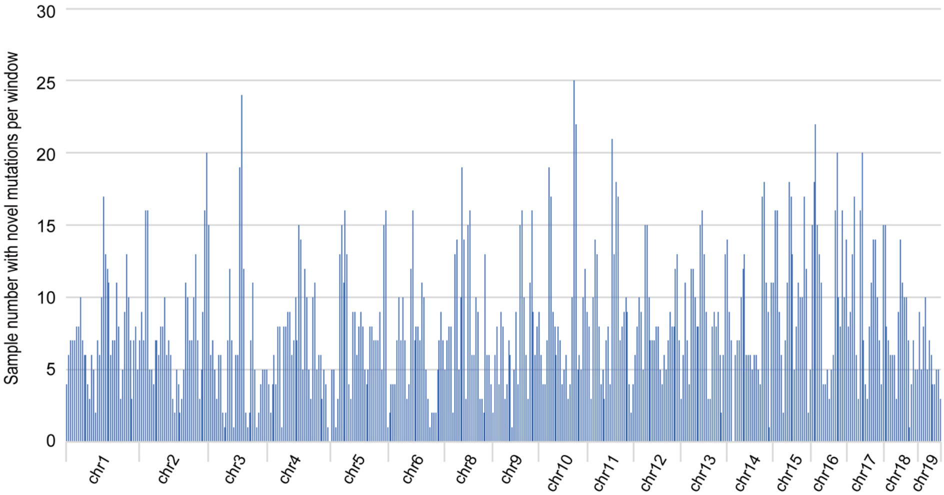
30
25
20
15
10
5
0
Sample number with novel mutations per window
chr1
chr2
chr3
chr4
chr5
chr6
chr8
chr9
chr10
chr11
chr12
chr13
chr14
chr15
chr16
chr17
chr18
chr19

b

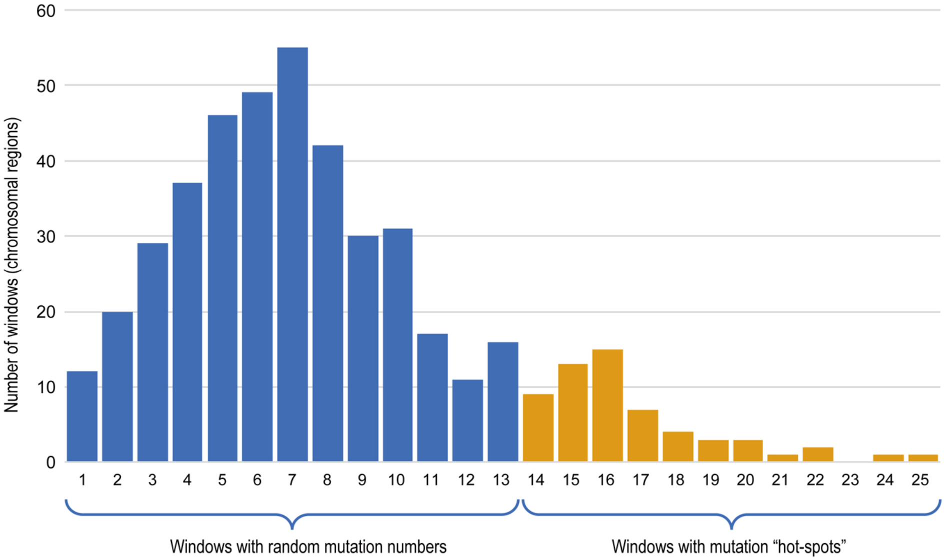
60
50
40
30
20
10
0
Number of windows (chromosomal regions)
1 2 3 4 5 6 7 8 9 10 11 12 13 14 15 16 17 18 19 20 21 22 23 24 25
Windows with random mutation numbers
Windows with mutation “hot-spots”

Figure S6

a. Gm5724

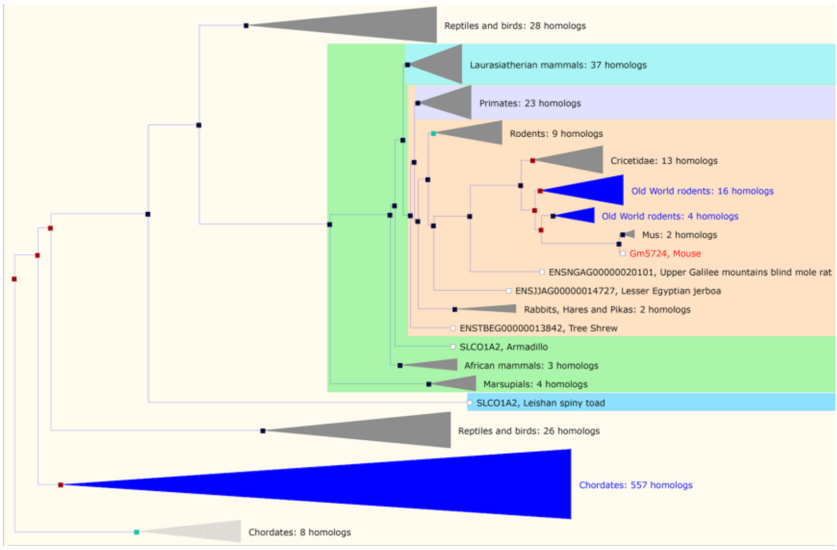

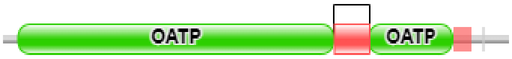

Source	Domain	Start	End
Pfam	OATP	21	597
transmembrane	n/a	21	40
transmembrane	n/a	60	80
transmembrane	n/a	87	109
transmembrane	n/a	157	182
transmembrane	n/a	194	222
transmembrane	n/a	242	266
disorder	n/a	276	293
low_complexity	n/a	283	298
coiled_coil	n/a	284	304
transmembrane	n/a	318	339
transmembrane	n/a	354	376
transmembrane	n/a	388	407
Pfam	Kazal_2	440	486
transmembrane	n/a	512	536
transmembrane	n/a	548	571
transmembrane	n/a	599	620
disorder	n/a	637	639

b. Gm35315

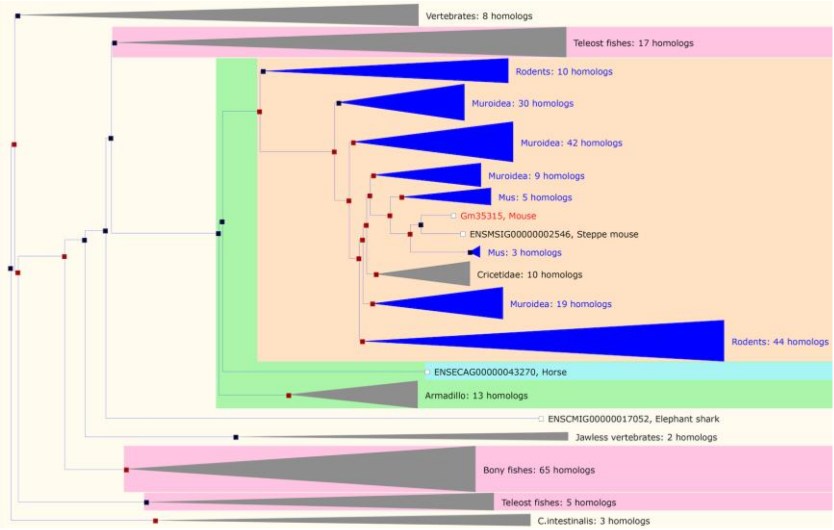

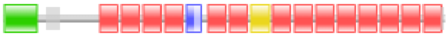

Source	Domain	Start	End
Pfam	KRAB	3	44
disorder	n/a	58	73
Pfam	zf-C2H2	126	148
Pfam	zf-C2H2	154	176
Pfam	zf-C2H2	182	204
Pfam	zf-C2H2	210	232
Pfam	zf-C2H2_6	238	256
Pfam	zf-C2H2	266	288
disorder	n/a	282	284
Pfam	zf-C2H2	294	316
Pfam	zf-C2H2_4	322	345
Pfam	zf-C2H2	350	372
Pfam	zf-C2H2	378	400
Pfam	zf-C2H2	406	428
Pfam	zf-C2H2	434	456
Pfam	zf-C2H2	462	484
disorder	n/a	478	480
disorder	n/a	482	483
Pfam	zf-C2H2	490	512
Pfam	zf-C2H2	518	540
Pfam	zf-C2H2	546	568
disorder	n/a	562	571

c. Gm3667

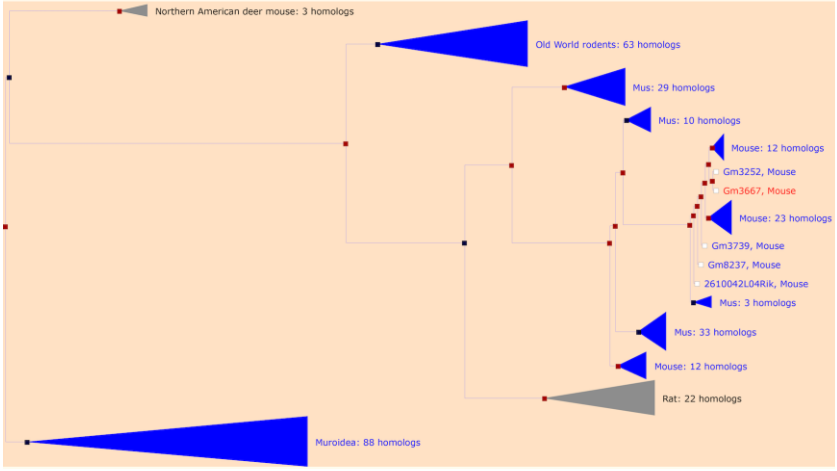

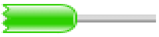

Source	Domain	Start	End
Pfam	Takusan	1	73

Figure S7

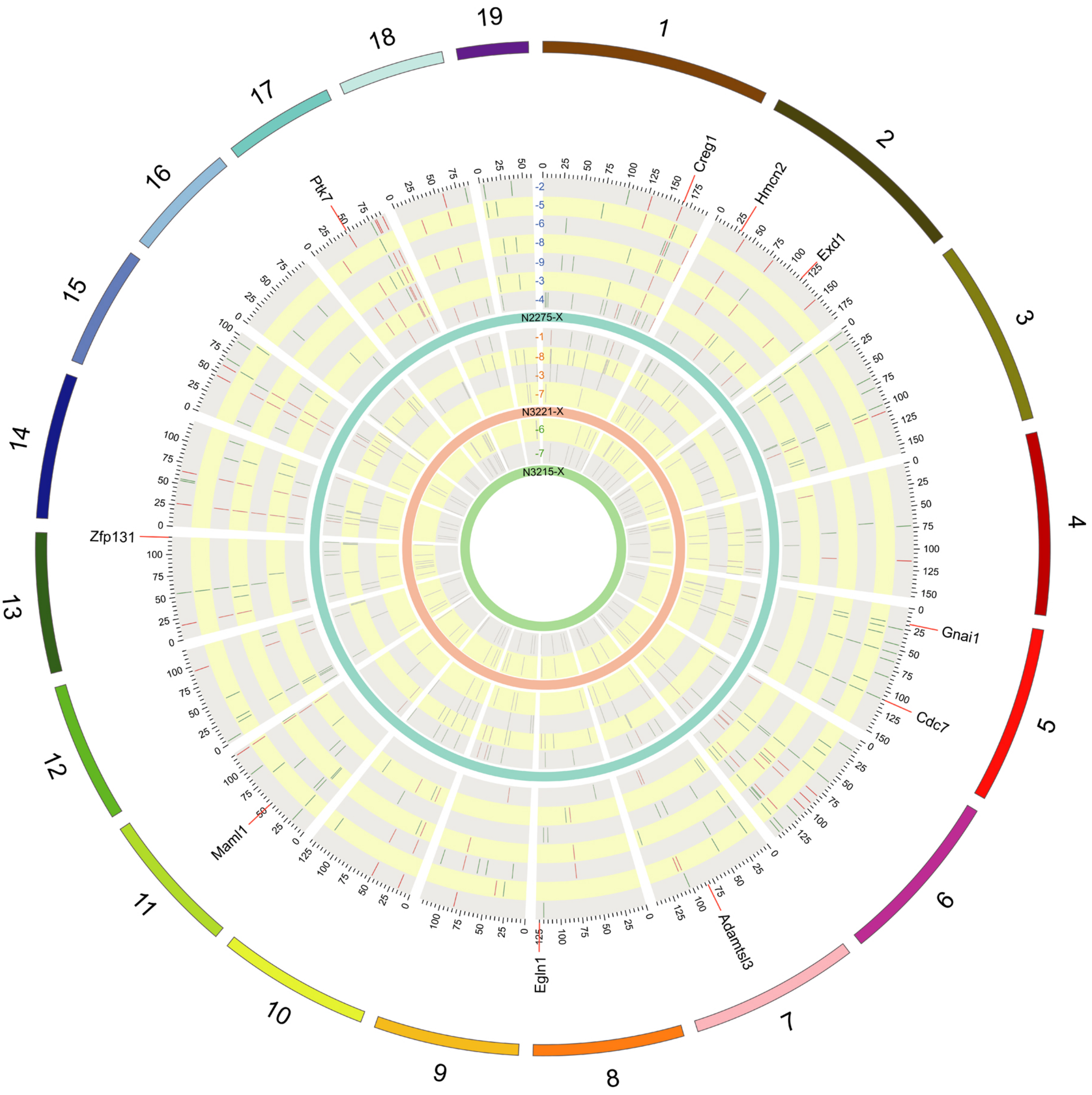

N2275-X
N3221-X
N3215-X
Creg1
Hmcn2
Exd1
Gnai1
Cdc7
Adamtsl3
Egln1
Maml1
Zfp131
Ptk7

Figure S8

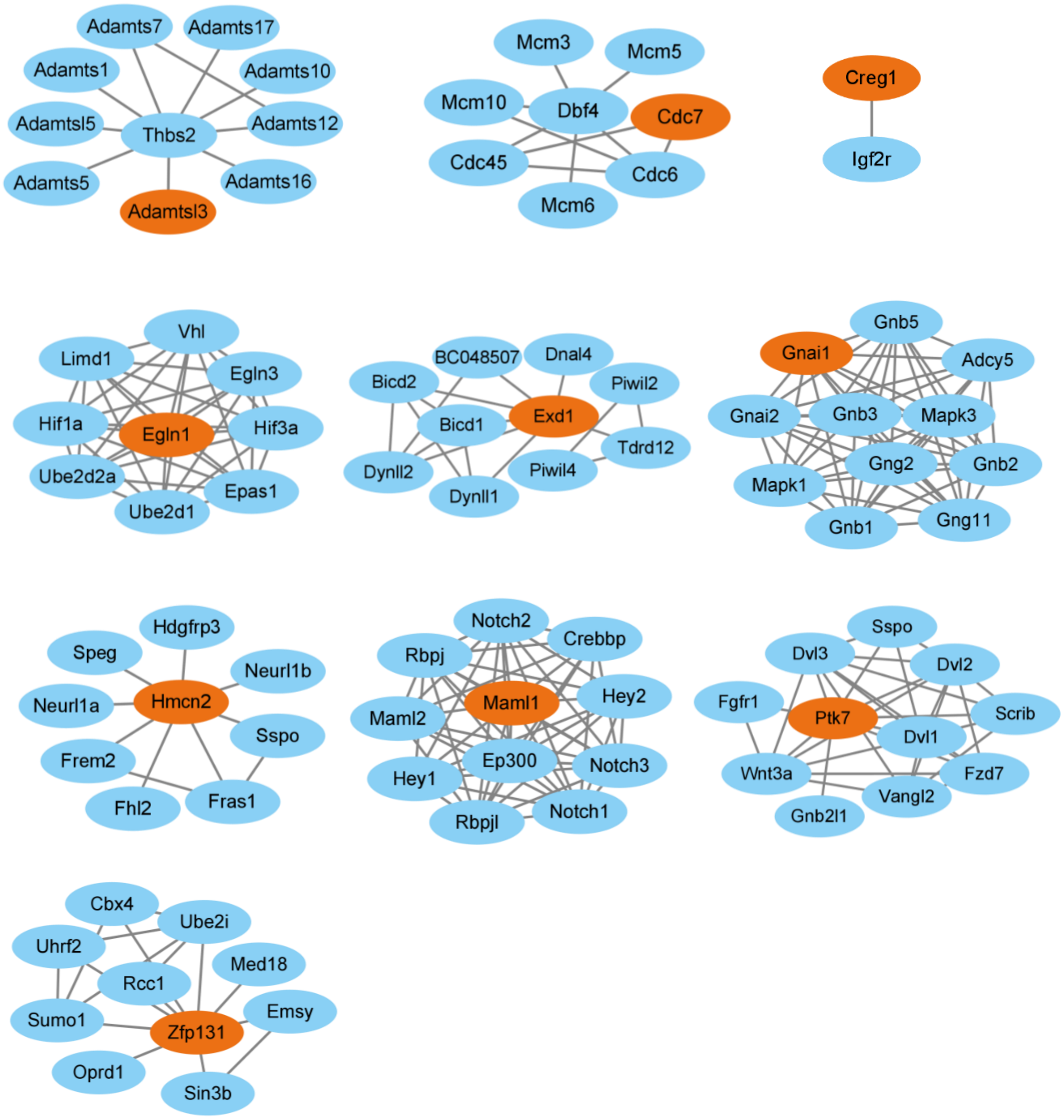
Adamts7
Adamts17
Adamts1
Adamts10
Adamtsl5
Thbs2
Adamts12
Adamts5
Adamts16
Adamtsl3
Mcm3
Mcm5
Mcm10
Dbf4
Cdc7
Cdc45
Cdc6
Mcm6
Creg1
Igf2r
Vhl
Limd1
Egln3
Hif1a
Egln1
Hif3a
Ube2d2a
Epas1
Ube2d1
BC048507
Dnal4
Bicd2
Piwil2
Exd1
Bicd1
Tdrd12
Dynll2
Piwil4
Dynll1
Gnb5
Gnai1
Adcy5
Gnai2
Gnb3
Mapk3
Gng2
Gnb2
Mapk1
Gnb1
Gng11
Hdgfrp3
Speg
Neurl1b
Neurl1a
Hmcn2
Sspo
Frem2
Fhl2
Fras1
Notch2
Crebbp
Rbpj
Hey2
Maml2
Maml1
Ep300
Notch3
Hey1
Notch1
Rbpjl
Sspo
Dvl3
Dvl2
Fgfr1
Scrib
Ptk7
Dvl1
Wnt3a
Fzd7
Vangl2
Gnb2l1
Cbx4
Ube2i
Uhrf2
Med18
Rcc1
Sumo1
Emsy
Zfp131
Oprd1
Sin3b

Figure S9

a. Deletion

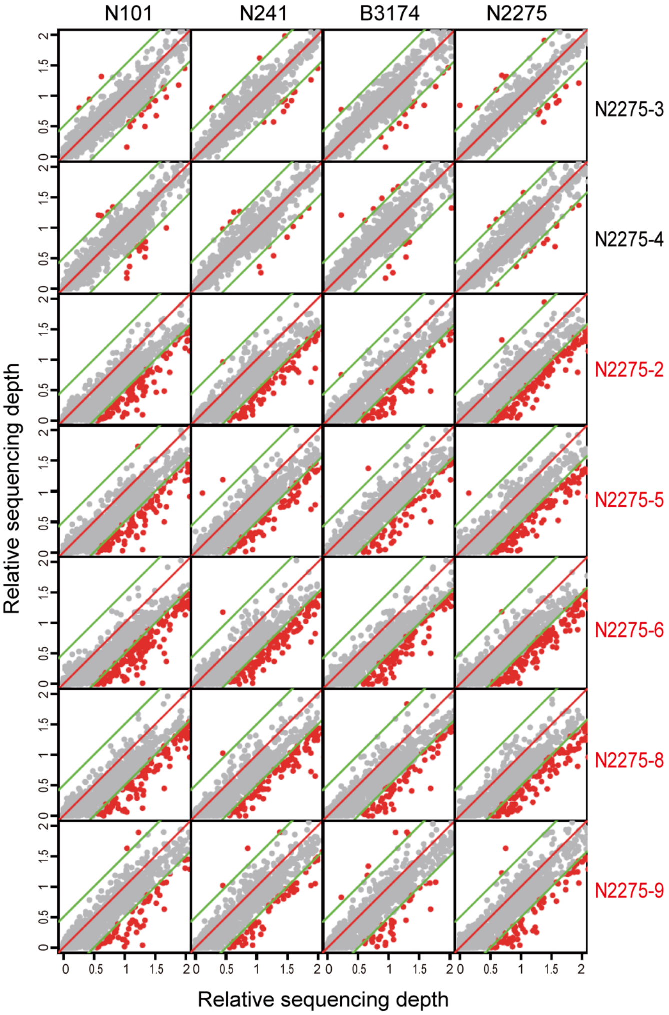

b. Duplication

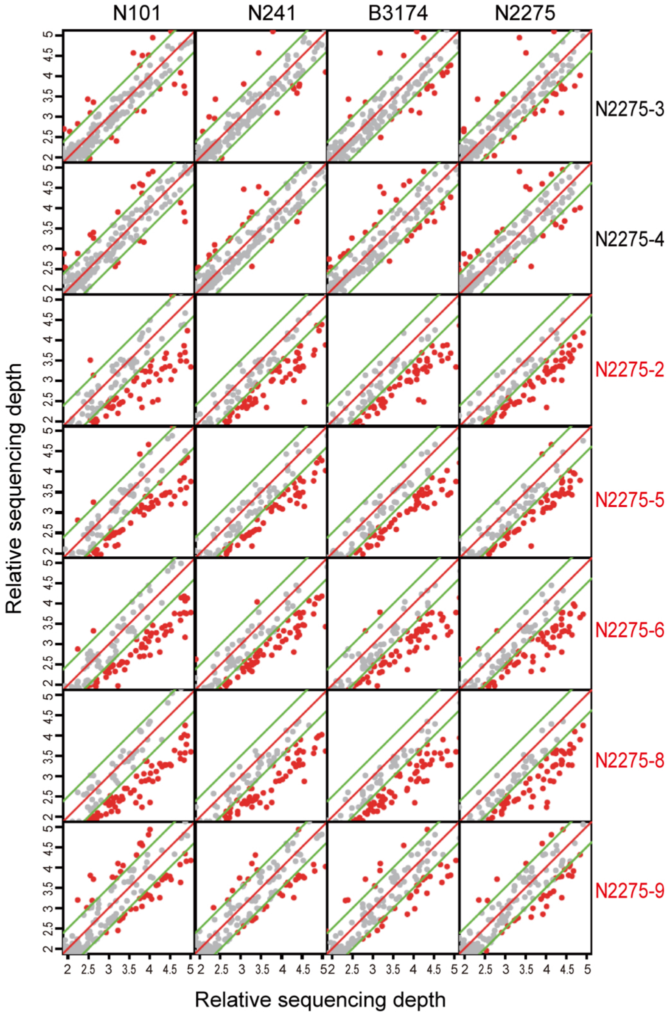

c. Distribution of transposable element types in deletion

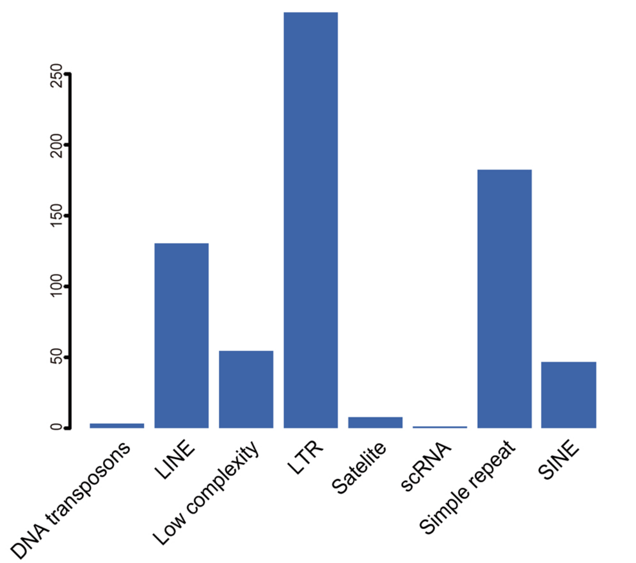

d. Distribution of transposable element types in duplication

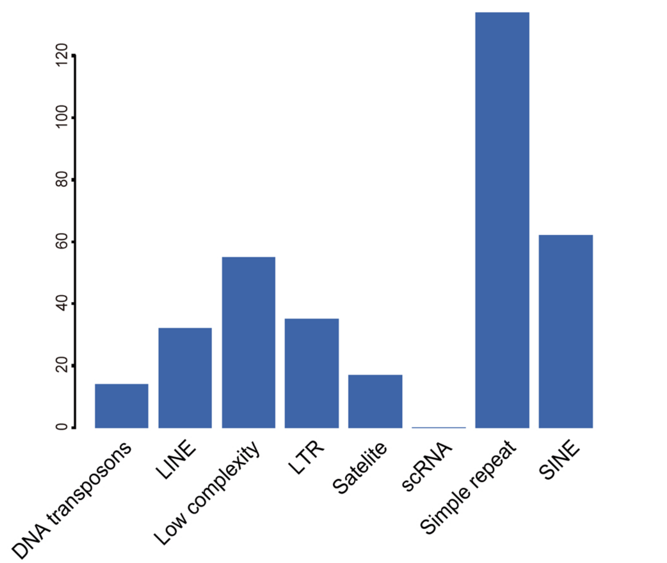

Figure S10

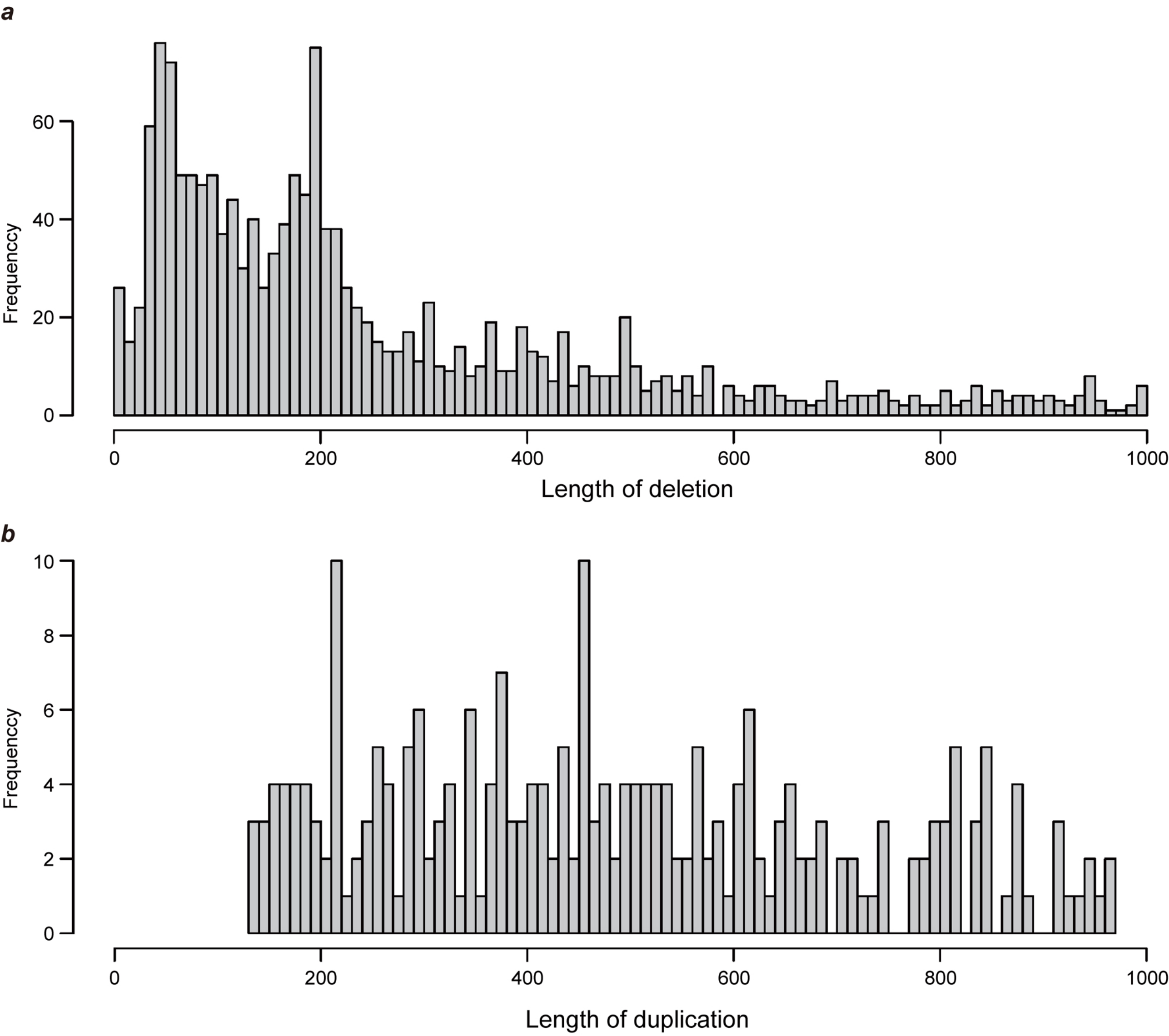
a
Frequency
Length of deletion
0
20
40
60
0
200
400
600
800
1000
b
Frequency
Length of duplication
0
2
4
6
8
10
0
200
400
600
800
1000

Sibship	Sample #	Chr	Length	#Reads	Coverage (bp)	Coverage (%)	Depth
Sibship C	C3108 (male)	chr1	195471971	52402824	191868150	98.1563	39.0365
		chr2	182113224	49743939	178211502	97.8575	39.5212
		chr3	160039680	41770440	156221352	97.6141	37.9895
		chr4	156508116	41150828	152015235	97.1293	38.1968
		chr5	151834684	40084113	147915253	97.4186	38.3231
		chr6	149736546	39088435	146221246	97.6523	38.0381
		chr7	145441459	38197448	141845997	97.5279	37.7842
		chr8	129401213	33787339	125486860	96.975	37.9991
		chr9	124595110	36242071	121131344	97.22	41.6905
		chr10	130694993	34316702	127052602	97.2131	38.1687
		chr11	122082543	32146409	118608342	97.1542	38.1551
		chr12	120129022	31949766	116920412	97.329	38.6356
		chr13	120421639	31391614	116884275	97.0625	37.9744
		chr14	124902244	33344530	121428210	97.2186	38.6002
		chr15	104043685	26755334	100640421	96.729	37.4783
		chr16	98207768	25752781	95018111	96.7521	37.9358
		chr17	94987271	25063108	91699423	96.5386	38.1862
		chr18	90702639	23260226	87405416	96.3648	37.3132
		chr19	61431566	15349084	58205187	94.748	36.3539
		chrX	171031299	22998072	163390432	95.5325	19.5069
		chrY	91744698	13207801	88080012	96.0056	20.9289
	N1240 (female)	chr1	195471971	67832269	191864117	98.15	37.42
		chr10	130694993	44597530	127050287	97.21	36.94
		chr11	122082543	41967078	118585401	97.14	36.99
		chr12	120129022	44088570	116919479	97.33	37.17
		chr13	120421639	41139462	116853504	97.04	36.95
		chr14	124902244	44353117	121425030	97.22	37.41
		chr15	104043685	35029721	100638305	96.73	36.33
		chr16	98207768	33431260	95017597	96.75	36.51
		chr17	94987271	32703811	91697857	96.54	36.92
		chr18	90702639	30317359	87393926	96.35	35.97
		chr19	61431566	20051954	58204554	94.75	35.1
		chr2	182113224	95001552	178192279	97.85	36.54
		chr3	160039680	54652534	156196379	97.6	36.73
		chr4	156508116	53577289	152007183	97.12	36.95
		chr5	151834684	52672300	147913789	97.42	37.52
		chr6	149736546	53203079	146216790	97.65	36.67
		chr7	145441459	49315818	141844856	97.53	36.49
		chr8	129401213	43998940	125467642	96.96	36.78
		chr9	124595110	68524830	121127265	97.22	37.15
		chrX	171031299	57115159	163393169	95.53	36.54
		chrY	91744698	496576	4023446	4.39	0.64
	N1240-2 (female)	chr1	195471971	51072665	191681461	98.0608	38.183
		chr2	182113224	62516980	178016529	97.7505	48.7147
		chr3	160039680	41371042	156027009	97.4927	37.7792
		chr4	156508116	39567568	151828987	97.0103	36.9535
		chr5	151834684	39095167	147756030	97.3138	37.622
		chr6	149736546	39014821	146062936	97.5466	37.9708
		chr7	145441459	37017398	141703153	97.4297	37.2132
		chr8	129401213	33402125	125326378	96.851	37.6907
		chr9	124595110	38658389	121019011	97.1298	45.2176
		chr10	130694993	33699678	126910966	97.1047	37.662
		chr11	122082543	30491663	118444737	97.0202	36.4307

		chr12	120129022	32791715	116812323	97.2391	39.7764
		chr13	120421639	31181574	116738843	96.9417	37.8263
		chr14	124902244	32512048	121309959	97.1239	38.0725
		chr15	104043685	26556618	100535073	96.6278	37.2728
		chr16	98207768	24954132	94919206	96.6514	37.1382
		chr17	94987271	23579334	91584039	96.4172	36.2793
		chr18	90702639	23166535	87304367	96.2534	37.2938
		chr19	61431566	15209281	58144526	94.6493	36.1497
		chrX	171031299	43185420	163237960	95.4433	37.0102
		chrY	91744698	267462	2855695	3.11265	0.433766
	**N1240-3 (female)**	chr1	195471971	60441700	191711402	98.0762	45.2618
		chr2	182113224	76556033	178052995	97.7705	60.1402
		chr3	160039680	49117527	156074750	97.5225	44.9283
		chr4	156508116	46727703	151867621	97.035	43.7104
		chr5	151834684	46074403	147782218	97.331	44.4076
		chr6	149736546	46187409	146091107	97.5654	45.0312
		chr7	145441459	43533641	141724801	97.4446	43.8307
		chr8	129401213	39364277	125359484	96.8766	44.4902
		chr9	124595110	47099679	121033823	97.1417	55.289
		chr10	130694993	39845194	126938694	97.1259	44.6028
		chr11	122082543	35806249	118472615	97.043	42.8487
		chr12	120129022	39228728	116828746	97.2527	47.6954
		chr13	120421639	36905263	116782753	96.9782	44.8432
		chr14	124902244	38499001	121328540	97.1388	45.161
		chr15	104043685	31354613	100552876	96.6449	44.0792
		chr16	98207768	29555106	94934666	96.6672	44.0587
		chr17	94987271	27786896	91599545	96.4335	42.8189
		chr18	90702639	27422447	87322563	96.2735	44.2193
		chr19	61431566	17926051	58154408	94.6654	42.6741
		chrX	171031299	51499483	163274750	95.4648	44.2066
		chrY	91744698	325078	3129776	3.4114	0.526909
**Sibship A**	**A3103 (male)**	chr1	195471971	69294669	191865141	98.15	39.46
		chr2	182113224	88382127	178193580	97.85	38.52
		chr3	160039680	55968781	156195749	97.6	38.83
		chr4	156508116	54464825	152006980	97.12	38.79
		chr5	151834684	53506907	147913902	97.42	39.34
		chr6	149736546	53978589	146216070	97.65	38.65
		chr7	145441459	49944928	141844726	97.53	38.12
		chr8	129401213	44736248	125469799	96.96	38.61
		chr9	124595110	63185698	121127016	97.22	38.81
		chr10	130694993	45435525	127051027	97.21	38.82
		chr11	122082543	42339798	118589238	97.14	38.54
		chr12	120129022	44070191	116919613	97.33	39.03
		chr13	120421639	41885202	116858349	97.04	38.82
		chr14	124902244	44893511	121426113	97.22	39.41
		chr15	104043685	35685632	100638791	96.73	38.2
		chr16	98207768	34189002	95017458	96.75	38.56
		chr17	94987271	33256478	91698426	96.54	38.78
		chr18	90702639	30898286	87394172	96.35	37.83
		chr19	61431566	20290671	58204664	94.75	36.67
		chrX	171031299	30083400	163382050	95.53	19.88
		chrY	91744698	14916187	88060416	95.98	21.37
	**N1241 (female)**	chr1	195471971	68677991	191864979	98.15	37.63
		chr2	182113224	92536589	178197716	97.85	36.84

		chr3	160039680	55272565	156200957	97.6	36.9
		chr4	156508116	54498351	152010058	97.13	37.32
		chr5	151834684	53546646	147914009	97.42	37.87
		chr6	149736546	53747259	146217349	97.65	36.95
		chr7	145441459	50214416	141845004	97.53	36.89
		chr8	129401213	44742590	125473304	96.96	37.13
		chr9	124595110	67074177	121129196	97.22	37.57
		chr10	130694993	45286500	127051045	97.21	37.25
		chr11	122082543	42987693	118594224	97.14	37.6
		chr12	120129022	44426346	116919925	97.33	37.39
		chr13	120421639	41758439	116859030	97.04	37.27
		chr14	124902244	44870593	121427043	97.22	37.61
		chr15	104043685	35583151	100638596	96.73	36.65
		chr16	98207768	33842470	95017468	96.75	36.72
		chr17	94987271	33339585	91698558	96.54	37.37
		chr18	90702639	30814697	87396811	96.36	36.31
		chr19	61431566	20478371	58204777	94.75	35.6
		chrX	171031299	57518921	163395137	95.54	36.57
		chrY	91744698	480478	3467134	3.78	0.61
	**N1241-2 (female)**	chr1	195471971	46414723	191865774	98.1551	35.0212
		chr2	182113224	43683738	178194365	97.8481	35.1401
		chr3	160039680	36937279	156194940	97.5976	34.0222
		chr4	156508116	36564723	152007306	97.1242	34.3613
		chr5	151834684	35554238	147914686	97.4182	34.4126
		chr6	149736546	34477243	146217990	97.6502	33.9981
		chr7	145441459	33921284	141846097	97.528	33.9948
		chr8	129401213	29925153	125467474	96.96	34.0918
		chr9	124595110	33196736	121129395	97.2184	38.4979
		chr10	130694993	30607870	127051154	97.212	34.4896
		chr11	122082543	28900479	118582503	97.1331	34.6999
		chr12	120129022	28341734	116920131	97.3288	34.6682
		chr13	120421639	27812196	116846899	97.0315	34.0706
		chr14	124902244	29584459	121425971	97.2168	34.6289
		chr15	104043685	23672812	100639223	96.7279	33.5904
		chr16	98207768	22834274	95018002	96.752	33.9973
		chr17	94987271	22473076	91699253	96.5385	34.6659
		chr18	90702639	20671963	87394369	96.3526	33.5798
		chr19	61431566	13677938	58204987	94.7477	32.8138
		chrX	171031299	39425876	163392633	95.5338	34.0538
		chrY	91744698	479251	3726423	4.06173	0.679809
	**N1241-5 (male)**	chr1	195471971	50939820	191866076	98.1553	38.5057
		chr2	182113224	47974267	178196189	97.8491	38.6608
		chr3	160039680	40635519	156197073	97.599	37.4954
		chr4	156508116	40160466	152007854	97.1246	37.8104
		chr5	151834684	39037460	147914995	97.4184	37.8734
		chr6	149736546	37957763	146218811	97.6507	37.4884
		chr7	145441459	37105561	141846057	97.5279	37.3506
		chr8	129401213	32872134	125468366	96.9607	37.5161
		chr9	124595110	36450735	121129092	97.2182	42.3234
		chr10	130694993	33558736	127051331	97.2121	37.876
		chr11	122082543	31710052	118586715	97.1365	38.1469
		chr12	120129022	31129071	116920324	97.329	38.1427
		chr13	120421639	30581095	116856202	97.0392	37.5262
		chr14	124902244	32444184	121427143	97.2177	38.0938

		chr15	104043685	26037971	100639479	96.7281	37.0076
		chr16	98207768	25042080	95018362	96.7524	37.4059
		chr17	94987271	24575387	91700143	96.5394	37.9842
		chr18	90702639	22701170	87396975	96.3555	36.9484
		chr19	61431566	15048167	58204973	94.7477	36.1557
		chrX	171031299	22612428	163385407	95.5295	19.4892
		chrY	91744698	12615532	88069437	95.994	20.2825
	**N1241-6 (female)**	chr1	195471971	62344422	191722687	98.0819	46.8136
		chr2	182113224	77373614	178050779	97.7693	60.7582
		chr3	160039680	49909175	156057456	97.5117	45.7703
		chr4	156508116	48738407	151871085	97.0372	45.7095
		chr5	151834684	48251691	147790981	97.3368	46.6287
		chr6	149736546	47674398	146095780	97.5686	46.6186
		chr7	145441459	45756786	141725913	97.4453	46.1923
		chr8	129401213	41228164	125360867	96.8777	46.7187
		chr9	124595110	47766993	121039976	97.1467	56.1071
		chr10	130694993	41366149	126936808	97.1245	46.4316
		chr11	122082543	38139314	118473944	97.0441	45.7626
		chr12	120129022	40173035	116832164	97.2556	48.9484
		chr13	120421639	38268982	116761336	96.9604	46.6243
		chr14	124902244	39654203	121333915	97.1431	46.6326
		chr15	104043685	32684545	100554833	96.6467	46.0771
		chr16	98207768	30445737	94938586	96.6712	45.5094
		chr17	94987271	29202206	91608163	96.4426	45.1198
		chr18	90702639	28444091	87321691	96.2725	45.9903
		chr19	61431566	18840875	58159039	94.6729	44.978
		chrX	171031299	51596325	163268711	95.4613	44.3941
		chrY	91744698	308654	2985295	3.25392	0.50113
	**N1241-7 (female)**	chr1	195471971	62731360	191716227	98.0786	47.1432
		chr2	182113224	79293344	178045967	97.7666	62.1661
		chr3	160039680	50817233	156057015	97.5115	46.6404
		chr4	156508116	48732713	151868341	97.0354	45.7406
		chr5	151834684	48200915	147786290	97.3337	46.6212
		chr6	149736546	47953693	146094330	97.5676	46.9188
		chr7	145441459	45649379	141727070	97.4461	46.1248
		chr8	129401213	41194711	125358749	96.876	46.7203
		chr9	124595110	48340369	121038855	97.1458	56.8051
		chr10	130694993	41518348	126936404	97.1242	46.6409
		chr11	122082543	37676045	118471243	97.0419	45.2444
		chr12	120129022	40501239	116833908	97.257	49.3788
		chr13	120421639	38311131	116758335	96.9579	46.715
		chr14	124902244	39985453	121328873	97.1391	47.0579
		chr15	104043685	32711095	100554838	96.6467	46.1524
		chr16	98207768	30697833	94939550	96.6721	45.9222
		chr17	94987271	29238180	91607051	96.4414	45.2137
		chr18	90702639	28507669	87325331	96.2765	46.1302
		chr19	61431566	18765986	58156181	94.6682	44.8366
		chrX	171031299	53009639	163274991	95.465	45.6446
		chrY	91744698	308403	2987818	3.25667	0.500707
	**N1241-8 (male)**	chr1	195471971	63117368	191725425	98.0833	47.2322
		chr2	182113224	79557186	178060557	97.7746	62.2248
		chr3	160039680	51127806	156070377	97.5198	46.7263
		chr4	156508116	48935381	151875414	97.04	45.7358
		chr5	151834684	48377424	147794154	97.3389	46.5926

		chr6	149736546	48187259	146101267	97.5722	46.9547
		chr7	145441459	45758055	141733414	97.4505	46.0363
		chr8	129401213	41363329	125361963	96.8785	46.7049
		chr9	124595110	48571191	121047052	97.1523	56.8582
		chr10	130694993	41732589	126945910	97.1314	46.6779
		chr11	122082543	37814790	118476379	97.0461	45.2083
		chr12	120129022	40666367	116842122	97.2639	49.3833
		chr13	120421639	38643224	116765680	96.964	46.9185
		chr14	124902244	40200189	121340724	97.1486	47.1129
		chr15	104043685	32908950	100560266	96.652	46.2299
		chr16	98207768	30877069	94944438	96.6771	45.9951
		chr17	94987271	29353897	91609364	96.4438	45.199
		chr18	90702639	28700749	87330488	96.2822	46.242
		chr19	61431566	18868171	58159246	94.6732	44.8862
		chrX	171031299	27356124	163148658	95.3911	23.4695
		chrY	91744698	12071332	85162569	92.8256	19.6281
**Sibship B-1**	**B3147((male)**	chr1	195471971	67787156	191865063	98.15	38.38
		chr2	182113224	85438031	178193140	97.85	37.76
		chr3	160039680	54479286	156196894	97.6	37.56
		chr4	156508116	53740104	152009315	97.13	38.07
		chr5	151834684	52852362	147913623	97.42	38.66
		chr6	149736546	52879452	146217083	97.65	37.7
		chr7	145441459	49598385	141844445	97.53	37.61
		chr8	129401213	44198785	125472152	96.96	37.95
		chr9	124595110	61504777	121128408	97.22	38.42
		chr10	130694993	44674026	127050662	97.21	37.95
		chr11	122082543	42602855	118590336	97.14	38.62
		chr12	120129022	43174137	116919808	97.33	38.12
		chr13	120421639	41267903	116853245	97.04	38.04
		chr14	124902244	43982103	121426550	97.22	38.37
		chr15	104043685	35257087	100638243	96.73	37.51
		chr16	98207768	33542578	95017296	96.75	37.61
		chr17	94987271	32888478	91698256	96.54	38.13
		chr18	90702639	30456523	87397110	96.36	37.07
		chr19	61431566	20337883	58204459	94.75	36.56
		chrX	171031299	29078941	163380324	95.53	19.09
		chrY	91744698	14315678	88060293	95.98	20.35
	**N2261 (female)**	chr1	195471971	52293019	191866216	98.1554	39.441
		chr2	182113224	49004781	178198245	97.8503	39.4528
		chr3	160039680	41681557	156206368	97.6048	38.3781
		chr4	156508116	41140647	152010751	97.1264	38.6505
		chr5	151834684	40046488	147913857	97.4177	38.7724
		chr6	149736546	38919052	146219462	97.6512	38.3556
		chr7	145441459	38006916	141846271	97.5281	38.1916
		chr8	129401213	33762581	125474865	96.9658	38.4469
		chr9	124595110	35954202	121130175	97.219	41.8472
		chr10	130694993	34284770	127051307	97.2121	38.6126
		chr11	122082543	32219353	118580486	97.1314	38.6919
		chr12	120129022	31786263	116920417	97.329	38.8973
		chr13	120421639	31242401	116864983	97.0465	38.2564
		chr14	124902244	33158137	121427234	97.2178	38.8937
		chr15	104043685	26629849	100639354	96.728	37.7652
		chr16	98207768	25625146	95018254	96.7523	38.2096
		chr17	94987271	25027167	91699568	96.5388	38.592

		chr18	90702639	23106241	87397169	96.3557	37.5245
		chr19	61431566	15277583	58205098	94.7479	36.6318
		chrX	171031299	43847585	163395887	95.5357	37.8517
		chrY	91744698	391544	3787895	4.12873	0.54093
	**N2261-2 (female)**	chr1	195471971	47571910	191862729	98.1536	35.8367
		chr2	182113224	44980582	178183726	97.8423	36.2663
		chr3	160039680	38322901	156183420	97.5904	35.2198
		chr4	156508116	38005269	151999821	97.1194	35.6722
		chr5	151834684	37274899	147913746	97.4176	36.1096
		chr6	149736546	35844004	146216704	97.6493	35.2482
		chr7	145441459	34862226	141844317	97.5267	35.1877
		chr8	129401213	31140609	125454000	96.9496	35.4036
		chr9	124595110	33964510	121125681	97.2154	39.6276
		chr10	130694993	31672447	127048584	97.21	35.6636
		chr11	122082543	30155464	118567619	97.1209	36.2273
		chr12	120129022	29462890	116918888	97.3278	35.9833
		chr13	120421639	29169181	116843912	97.029	35.6368
		chr14	124902244	30696577	121423630	97.2149	36.1072
		chr15	104043685	24793542	100637382	96.7261	35.0818
		chr16	98207768	23484051	95016924	96.7509	35.1297
		chr17	94987271	23183487	91698159	96.5373	35.7405
		chr18	90702639	21488442	87388223	96.3458	34.8308
		chr19	61431566	14292733	58204421	94.7468	34.1921
		chrX	171031299	40555209	163392703	95.5338	34.9108
		chrY	91744698	385254	3218324	3.50791	0.570663
	**N2261-3 (female)**	chr1	195471971	39076052	191734568	98.088	28.9447
		chr2	182113224	39029658	178070275	97.78	30.4794
		chr3	160039680	31322372	156078982	97.5252	28.2788
		chr4	156508116	31059296	151891718	97.0504	28.6089
		chr5	151834684	30055698	147802035	97.3441	28.6123
		chr6	149736546	29411860	146109837	97.5779	28.4061
		chr7	145441459	28414672	141746761	97.4597	28.1505
		chr8	129401213	25450524	125373701	96.8876	28.4351
		chr9	124595110	29765900	121049429	97.1542	33.4085
		chr10	130694993	25806471	126954146	97.1377	28.5598
		chr11	122082543	24705274	118504051	97.0688	29.1284
		chr12	120129022	24228814	116840676	97.2627	29.0398
		chr13	120421639	23733975	116760040	96.9594	28.4884
		chr14	124902244	24942389	121340314	97.1482	28.7146
		chr15	104043685	20158605	100560208	96.6519	28.0314
		chr16	98207768	19223523	94950697	96.6835	28.226
		chr17	94987271	19143067	91631741	96.4674	28.9569
		chr18	90702639	17614983	87334317	96.2864	28.0577
		chr19	61431566	11734302	58163596	94.6803	27.5844
		chrX	171031299	32380481	163269351	95.4617	27.3978
		chrY	91744698	358119	5049984	5.50439	0.480687
	**N2261-4 (male)**	chr1	195471971	44424585	191769002	98.1056	32.9627
		chr2	182113224	44276998	178111217	97.8025	34.6589
		chr3	160039680	35597133	156119578	97.5505	32.1994
		chr4	156508116	35364395	151922065	97.0698	32.6382
		chr5	151834684	34238268	147827706	97.361	32.6469
		chr6	149736546	33509773	146141580	97.5991	32.42
		chr7	145441459	32327797	141771442	97.4766	32.0867
		chr8	129401213	28941722	125400485	96.9083	32.3879

		chr9	124595110	33715724	121068713	97.1697	37.9243
		chr10	130694993	29368272	126980304	97.1577	32.5573
		chr11	122082543	28176183	118531527	97.0913	33.2803
		chr12	120129022	27530112	116861323	97.2798	33.0607
		chr13	120421639	27004008	116791634	96.9856	32.4729
		chr14	124902244	28387363	121362832	97.1663	32.7369
		chr15	104043685	22945973	100583678	96.6745	31.9617
		chr16	98207768	21867747	94966570	96.6997	32.1698
		chr17	94987271	21682402	91649083	96.4856	32.8605
		chr18	90702639	20045765	87354951	96.3092	31.9871
		chr19	61431566	13413004	58174239	94.6976	31.5827
		chrX	171031299	19284237	163178846	95.4088	16.2739
		chrY	91744698	9583486	87983874	95.9008	15.0748
	**N2261-5 (male)**	chr1	195471971	37496255	191749777	98.0958	28.1308
		chr2	182113224	36760053	178069701	97.7797	29.2282
		chr3	160039680	29970616	156074013	97.5221	27.4108
		chr4	156508116	29861862	151897427	97.054	27.8745
		chr5	151834684	28929492	147817396	97.3542	27.9011
		chr6	149736546	28241960	146123265	97.5869	27.6377
		chr7	145441459	27375735	141757004	97.4667	27.474
		chr8	129401213	24455746	125368017	96.8832	27.6805
		chr9	124595110	27825963	121056553	97.16	31.8867
		chr10	130694993	24777391	126963318	97.1447	27.7715
		chr11	122082543	23887753	118497400	97.0633	28.5403
		chr12	120129022	23176494	116851141	97.2714	28.1584
		chr13	120421639	22819577	116744677	96.9466	27.7481
		chr14	124902244	23834217	121350766	97.1566	27.8324
		chr15	104043685	19381712	100573073	96.6643	27.3033
		chr16	98207768	18431855	94959395	96.6923	27.4105
		chr17	94987271	18424978	91639799	96.4759	28.2411
		chr18	90702639	16930268	87332095	96.284	27.3157
		chr19	61431566	11325651	58171024	94.6924	26.9736
		chrX	171031299	16237501	163125841	95.3778	13.8647
		chrY	91744698	8102648	87938410	95.8512	12.8861
	**N2261-6 (female)**	chr1	195471971	40015442	191738122	98.0898	29.9135
		chr2	182113224	39405256	178066004	97.7776	31.1436
		chr3	160039680	31975342	156077633	97.5243	29.1368
		chr4	156508116	31810036	151895015	97.0525	29.5737
		chr5	151834684	30797070	147803375	97.3449	29.5889
		chr6	149736546	30109916	146114863	97.5813	29.356
		chr7	145441459	29216414	141744915	97.4584	29.2153
		chr8	129401213	26089117	125365449	96.8812	29.4193
		chr9	124595110	29973036	121049654	97.1544	34.0795
		chr10	130694993	26327067	126953929	97.1376	29.4044
		chr11	122082543	25286806	118502398	97.0674	30.0916
		chr12	120129022	24789231	116845225	97.2664	29.9931
		chr13	120421639	24241409	116748549	96.9498	29.3693
		chr14	124902244	25510268	121343820	97.151	29.6611
		chr15	104043685	20607944	100566252	96.6577	28.9247
		chr16	98207768	19640149	94950996	96.6838	29.103
		chr17	94987271	19489527	91631125	96.4667	29.752
		chr18	90702639	17988750	87333566	96.2856	28.9223
		chr19	61431566	12008564	58165932	94.6841	28.4952
		chrX	171031299	33051637	163269538	95.4618	28.2281

		chrY	91744698	373681	5451286	5.9418	0.516442
	**N2261-8 (male)**	chr1	195471971	44674489	191861422	98.1529	33.2165
		chr2	182113224	42257330	178185872	97.8435	33.6289
		chr3	160039680	35988124	156181424	97.5892	32.6494
		chr4	156508116	35623676	151999808	97.1194	33.0025
		chr5	151834684	34903658	147912570	97.4169	33.369
		chr6	149736546	33672356	146214659	97.6479	32.6793
		chr7	145441459	32686641	141843840	97.5264	32.5299
		chr8	129401213	29151848	125452327	96.9483	32.7073
		chr9	124595110	31999713	121125052	97.2149	36.8567
		chr10	130694993	29732236	127047962	97.2095	33.037
		chr11	122082543	28268413	118571547	97.1241	33.5155
		chr12	120129022	27612675	116918651	97.3276	33.292
		chr13	120421639	27438248	116840154	97.0259	33.0929
		chr14	124902244	28837982	121423608	97.2149	33.45
		chr15	104043685	23258426	100637175	96.7259	32.4806
		chr16	98207768	22044658	95017371	96.7514	32.5516
		chr17	94987271	21796281	91697977	96.5371	33.1763
		chr18	90702639	20184254	87386824	96.3443	32.2948
		chr19	61431566	13456682	58204171	94.7464	31.7695
		chrX	171031299	19878631	163374256	95.523	16.8589
		chrY	91744698	10832760	88046049	95.9685	17.1132
**Sibship B-2**	**B3142 (male)**	chr1	195471971	64326128	191863645	98.15	35.89
		chr2	182113224	83211123	178196911	97.85	35.1
		chr3	160039680	51703735	156203554	97.6	35.14
		chr4	156508116	50842652	152010473	97.13	35.49
		chr5	151834684	50014300	147913783	97.42	36
		chr6	149736546	50224508	146217093	97.65	35.16
		chr7	145441459	47575460	141843948	97.53	35.14
		chr8	129401213	41788463	125475674	96.97	35.35
		chr9	124595110	60664697	121127915	97.22	35.75
		chr10	130694993	42234731	127051647	97.21	35.35
		chr11	122082543	39885475	118595865	97.14	35.6
		chr12	120129022	40954490	116918653	97.33	35.54
		chr13	120421639	38900068	116863076	97.04	35.36
		chr14	124902244	41817334	121425280	97.22	35.73
		chr15	104043685	33190232	100638703	96.73	34.85
		chr16	98207768	31579037	95017006	96.75	34.89
		chr17	94987271	31049982	91698127	96.54	35.48
		chr18	90702639	28751977	87399875	96.36	34.5
		chr19	61431566	19007840	58204014	94.75	33.69
		chrX	171031299	27956701	163381806	95.53	18.08
		chrY	91744698	13638972	88055713	95.98	19.13
	**N2293 (female)**	chr1	195471971	51565783	191865844	98.1552	38.8566
		chr2	182113224	48448299	178200315	97.8514	38.9482
		chr3	160039680	41006005	156201244	97.6016	37.719
		chr4	156508116	40681745	152009423	97.1256	38.1828
		chr5	151834684	39659604	147914587	97.4182	38.3581
		chr6	149736546	38434908	146218586	97.6506	37.844
		chr7	145441459	37688473	141846380	97.5282	37.8547
		chr8	129401213	33385787	125475738	96.9664	37.9833
		chr9	124595110	35890425	121130467	97.2193	41.6885
		chr10	130694993	33858470	127052387	97.2129	38.0933
		chr11	122082543	32022746	118589715	97.139	38.4169

		chr12	120129022	31471935	116920606	97.3292	38.4742
		chr13	120421639	30854709	116793559	96.9872	37.745
		chr14	124902244	32754001	121426269	97.217	38.3667
		chr15	104043685	26333412	100639614	96.7282	37.31
		chr16	98207768	25287423	95018420	96.7524	37.6491
		chr17	94987271	24860627	91699896	96.5391	38.3013
		chr18	90702639	22834202	87398086	96.3567	37.0425
		chr19	61431566	15149834	58205137	94.7479	36.288
		chrX	171031299	41881010	163397308	95.5365	36.1168
		chrY	91744698	423310	3762677	4.10125	0.588254
	**N2293-1 (male)**	chr1	195471971	41564061	191686969	98.0637	31.4585
		chr2	182113224	57373596	178000069	97.7414	45.7127
		chr3	160039680	33514424	155986044	97.4671	30.9716
		chr4	156508116	32677879	151833106	97.0129	30.8818
		chr5	151834684	32361423	147764507	97.3193	31.522
		chr6	149736546	31884355	146073774	97.5539	31.4333
		chr7	145441459	30676877	141708141	97.4331	31.2113
		chr8	129401213	27584916	125311316	96.8394	31.4974
		chr9	124595110	34475081	121023453	97.1334	40.843
		chr10	130694993	27652851	126914654	97.1075	31.2885
		chr11	122082543	25864607	118416646	96.9972	31.2699
		chr12	120129022	27242286	116814408	97.2408	33.4549
		chr13	120421639	25675735	116680890	96.8936	31.5297
		chr14	124902244	26459896	121313470	97.1267	31.3541
		chr15	104043685	21871775	100538196	96.6308	31.0801
		chr16	98207768	20293899	94923642	96.6559	30.5743
		chr17	94987271	19645527	91592248	96.4258	30.5932
		chr18	90702639	19036462	87296952	96.2452	31.0219
		chr19	61431566	12742968	58149914	94.658	30.6713
		chrX	171031299	17822250	163022587	95.3174	15.4475
		chrY	91744698	7974110	87835864	95.7394	13.0068
	**N2293-2 (male)**	chr1	195471971	40921377	191656050	98.0478	30.9283
		chr2	182113224	54677447	177973588	97.7269	43.513
		chr3	160039680	32863837	155971188	97.4578	30.3289
		chr4	156508116	32364078	151808071	96.9969	30.5432
		chr5	151834684	32121440	147744951	97.3065	31.2446
		chr6	149736546	31457288	146050335	97.5382	30.9723
		chr7	145441459	30512420	141683160	97.4159	31.001
		chr8	129401213	27326090	125291397	96.824	31.1588
		chr9	124595110	33404004	121011271	97.1236	39.5289
		chr10	130694993	27323022	126898366	97.095	30.8712
		chr11	122082543	25977481	118405845	96.9884	31.3632
		chr12	120129022	26646657	116800376	97.2291	32.6834
		chr13	120421639	25448940	116670860	96.8853	31.2088
		chr14	124902244	25958546	121295350	97.1122	30.7197
		chr15	104043685	21661961	100525922	96.619	30.7379
		chr16	98207768	19964566	94909671	96.6417	30.0362
		chr17	94987271	19535632	91579523	96.4124	30.3823
		chr18	90702639	18829060	87285376	96.2325	30.6406
		chr19	61431566	12746613	58140577	94.6428	30.6368
		chrX	171031299	17288444	162958387	95.2799	14.9661
		chrY	91744698	8530924	87854617	95.7599	13.9115
	**N2293-5 (male)**	chr1	195471971	47304243	191865169	98.1548	35.6744
		chr2	182113224	44842381	178195597	97.8488	36.0302

		chr3	160039680	37675554	156197052	97.599	34.6859
		chr4	156508116	37444082	152006576	97.1238	35.1671
		chr5	151834684	36423578	147914726	97.4183	35.2287
		chr6	149736546	35282458	146218771	97.6507	34.769
		chr7	145441459	34905142	141846731	97.5284	34.8395
		chr8	129401213	30653515	125469597	96.9617	34.9001
		chr9	124595110	34289868	121128008	97.2173	39.6601
		chr10	130694993	31196986	127051138	97.2119	35.128
		chr11	122082543	29690632	118586368	97.1362	35.6304
		chr12	120129022	28963685	116920356	97.329	35.4066
		chr13	120421639	28378504	116856918	97.0398	34.7425
		chr14	124902244	30234364	121427435	97.218	35.3399
		chr15	104043685	24191755	100639239	96.7279	34.3059
		chr16	98207768	23289912	95018351	96.7524	34.6682
		chr17	94987271	22929459	91699100	96.5383	35.3394
		chr18	90702639	21115526	87396366	96.3548	34.2813
		chr19	61431566	14033519	58205368	94.7483	33.642
		chrX	171031299	20941084	163386625	95.5302	18.0071
		chrY	91744698	11899659	88067686	95.9921	19.1007
	**N2293-6 (female)**	chr1	195471971	50274284	191865671	98.1551	37.994
		chr2	182113224	47605774	178195974	97.849	38.3104
		chr3	160039680	40197516	156200484	97.6011	37.0842
		chr4	156508116	39670166	152007862	97.1246	37.3299
		chr5	151834684	38545115	147914743	97.4183	37.3674
		chr6	149736546	37479930	146218483	97.6505	37.01
		chr7	145441459	36717161	141846438	97.5282	36.8084
		chr8	129401213	32475453	125468506	96.9608	37.0501
		chr9	124595110	36347664	121129308	97.2183	42.057
		chr10	130694993	33161739	127050853	97.2117	37.421
		chr11	122082543	31334909	118585004	97.1351	37.677
		chr12	120129022	30750928	116920029	97.3287	37.6589
		chr13	120421639	30178246	116859257	97.0417	37.0208
		chr14	124902244	32079006	121426404	97.2172	37.5942
		chr15	104043685	25710573	100638735	96.7274	36.5335
		chr16	98207768	24813560	95018304	96.7523	37.0269
		chr17	94987271	24238455	91699642	96.5389	37.4397
		chr18	90702639	22464239	87395229	96.3536	36.5504
		chr19	61431566	14857080	58205312	94.7482	35.6978
		chrX	171031299	42527692	163394268	95.5347	36.7859
		chrY	91744698	429914	4103067	4.47227	0.598956
**Sibship B-3**	**N241 (female)**	chr1	195471971	52403593	191866853	98.1557	39.5538
		chr2	182113224	48987641	178199713	97.8511	39.4497
		chr3	160039680	41827338	156200197	97.6009	38.5386
		chr4	156508116	41010425	152009493	97.1256	38.5562
		chr5	151834684	39973707	147914862	97.4184	38.7275
		chr6	149736546	38962349	146219473	97.6512	38.4257
		chr7	145441459	37781978	141846538	97.5283	37.9958
		chr8	129401213	33651080	125473158	96.9644	38.3454
		chr9	124595110	35949058	121130799	97.2195	41.8273
		chr10	130694993	34291755	127051582	97.2123	38.6471
		chr11	122082543	32000296	118588083	97.1376	38.4562
		chr12	120129022	31838378	116920459	97.3291	38.9876
		chr13	120421639	31240604	116864310	97.0459	38.2756
		chr14	124902244	33236853	121427189	97.2178	39.0038

		chr15	104043685	26640272	100639001	96.7276	37.8043
		chr16	98207768	25686489	95018239	96.7523	38.3252
		chr17	94987271	24959839	91699691	96.5389	38.5139
		chr18	90702639	23150546	87398079	96.3567	37.6171
		chr19	61431566	15232017	58205298	94.7482	36.5452
		chrX	171031299	44207685	163396764	95.5362	38.1901
		chrY	91744698	414460	3994042	4.35343	0.577207
	**N101((male)**	chr1	195471971	69536534	191864921	98.15	39.47
		chr2	182113224	85958856	178194437	97.85	38.78
		chr3	160039680	55886401	156194875	97.6	38.64
		chr4	156508116	55053090	152009199	97.13	39.08
		chr5	151834684	54126432	147913495	97.42	39.67
		chr6	149736546	54068094	146217159	97.65	38.77
		chr7	145441459	50846422	141844568	97.53	38.57
		chr8	129401213	45288694	125471141	96.96	38.95
		chr9	124595110	62156439	121128802	97.22	39.49
		chr10	130694993	45889606	127051294	97.21	39.08
		chr11	122082543	43620726	118589464	97.14	39.6
		chr12	120129022	44141865	116919827	97.33	39.19
		chr13	120421639	42409038	116855800	97.04	39.19
		chr14	124902244	44969767	121425387	97.22	39.41
		chr15	104043685	36109535	100639114	96.73	38.53
		chr16	98207768	34408780	95017445	96.75	38.67
		chr17	94987271	33902745	91698364	96.54	39.42
		chr18	90702639	31313521	87396539	96.36	38.22
		chr19	61431566	20870113	58204594	94.75	37.63
		chrX	171031299	29813299	163383606	95.53	19.63
		chrY	91744698	14608409	88062003	95.99	20.83
	**N2275 (female)**	chr1	195471971	68403552	191863444	98.15	38.14
		chr2	182113224	92654228	178192207	97.85	37.35
		chr3	160039680	55075258	156187958	97.59	37.4
		chr4	156508116	54246858	152007424	97.12	37.79
		chr5	151834684	53298090	147913209	97.42	38.34
		chr6	149736546	53594623	146216453	97.65	37.41
		chr7	145441459	50087471	141844340	97.53	37.4
		chr8	129401213	44583858	125468817	96.96	37.64
		chr9	124595110	67556107	121126602	97.22	37.89
		chr10	130694993	45019615	127050397	97.21	37.66
		chr11	122082543	42544969	118585289	97.14	37.83
		chr12	120129022	43920967	116919679	97.33	37.9
		chr13	120421639	41506487	116844290	97.03	37.67
		chr14	124902244	44377125	121425930	97.22	38.12
		chr15	104043685	35361410	100637994	96.73	37.05
		chr16	98207768	33646332	95017252	96.75	37.13
		chr17	94987271	33087567	91698327	96.54	37.7
		chr18	90702639	30538117	87394473	96.35	36.59
		chr19	61431566	20233187	58204283	94.75	35.75
		chrX	171031299	57060421	163393085	95.53	36.91
		chrY	91744698	476249	3319029	3.62	0.63
	**B3174 (male)**	chr1	195471971	51851723	191866312	98.1554	38.9091
		chr2	182113224	48959080	178202530	97.8526	39.2283
		chr3	160039680	41295899	156207530	97.6055	37.8312
		chr4	156508116	40961947	152012557	97.1276	38.2871
		chr5	151834684	40073334	147914951	97.4184	38.5419

		chr6	149736546	38801100	146219849	97.6514	38.0431
		chr7	145441459	38237024	141846460	97.5282	37.9985
		chr8	129401213	33713259	125477841	96.9681	38.1955
		chr9	124595110	35665145	121131406	97.22	41.426
		chr10	130694993	34150674	127052151	97.2127	38.2413
		chr11	122082543	32461309	118593831	97.1423	38.8086
		chr12	120129022	31671427	116920480	97.3291	38.59
		chr13	120421639	31165392	116869721	97.0504	37.9753
		chr14	124902244	33089434	121428199	97.2186	38.539
		chr15	104043685	26671294	100640022	96.7286	37.6372
		chr16	98207768	25589059	95018321	96.7523	37.9604
		chr17	94987271	25241568	91699840	96.5391	38.7225
		chr18	90702639	23105620	87399953	96.3588	37.3349
		chr19	61431566	15412115	58205224	94.7481	36.7728
		chrX	171031299	22357269	163386978	95.5305	19.1183
		chrY	91744698	12665628	88077375	96.0027	20.2308
	**N2275-2 (female)**	chr1	195471971	41417820	191726754	98.084	31.1778
		chr2	182113224	40430323	178053911	97.771	32.282
		chr3	160039680	33031679	156057945	97.512	30.3102
		chr4	156508116	33065371	151884012	97.0455	30.9635
		chr5	151834684	31989675	147796042	97.3401	30.9576
		chr6	149736546	31124727	146103408	97.5736	30.5663
		chr7	145441459	30383648	141739974	97.455	30.6049
		chr8	129401213	27083690	125355620	96.8736	30.7597
		chr9	124595110	30618400	121043871	97.1498	35.2221
		chr10	130694993	27331727	126947282	97.1325	30.7401
		chr11	122082543	26465769	118492645	97.0595	31.7331
		chr12	120129022	25633522	116837902	97.2603	31.2459
		chr13	120421639	25157369	116734382	96.938	30.6969
		chr14	124902244	26310937	121336612	97.1453	30.8345
		chr15	104043685	21394813	100557459	96.6493	30.2451
		chr16	98207768	20334228	94947313	96.68	30.3446
		chr17	94987271	20469072	91628632	96.4641	31.4746
		chr18	90702639	18658168	87324101	96.2751	30.2145
		chr19	61431566	12504800	58160664	94.6755	29.8906
		chrX	171031299	34027161	163264184	95.4587	29.2614
		chrY	91744698	338057	5483747	5.97718	0.468763
	**N2275-3 (female)**	chr1	195471971	48514653	191861895	98.1531	36.0728
		chr2	182113224	45602893	178185932	97.8435	36.2802
		chr3	160039680	39151971	156181665	97.5893	35.5332
		chr4	156508116	38596515	152002490	97.1212	35.7272
		chr5	151834684	37810861	147912986	97.4171	36.1187
		chr6	149736546	36524049	146215124	97.6483	35.4431
		chr7	145441459	35406454	141844187	97.5267	35.2006
		chr8	129401213	31605761	125453455	96.9492	35.4347
		chr9	124595110	34425057	121124090	97.2142	39.6061
		chr10	130694993	32162576	127048598	97.21	35.7367
		chr11	122082543	30306687	118571414	97.124	35.8754
		chr12	120129022	30037646	116919173	97.328	36.1969
		chr13	120421639	29623321	116849432	97.0336	35.7196
		chr14	124902244	31319459	121423951	97.2152	36.3616
		chr15	104043685	25103279	100637393	96.7261	35.0429
		chr16	98207768	23919471	95017155	96.7512	35.3255
		chr17	94987271	23599350	91698222	96.5374	35.8721

		chr18	90702639	21817285	87386253	96.3437	34.9052
		chr19	61431566	14435795	58204305	94.7466	34.0572
		chrX	171031299	41770000	163393377	95.5342	35.5296
		chrY	91744698	381453	3227286	3.51768	0.557318
	**N2275-4 (female)**	chr1	195471971	49876001	191862660	98.1535	37.5209
		chr2	182113224	46922441	178186670	97.8439	37.7842
		chr3	160039680	40203937	156182369	97.5898	36.9045
		chr4	156508116	39747847	152001859	97.1208	37.2474
		chr5	151834684	38931966	147913277	97.4173	37.6539
		chr6	149736546	37536470	146216551	97.6492	36.8583
		chr7	145441459	36444453	141844271	97.5267	36.7096
		chr8	129401213	32566895	125453500	96.9492	36.9649
		chr9	124595110	35288475	121125698	97.2155	41.126
		chr10	130694993	33148834	127048471	97.2099	37.275
		chr11	122082543	31419592	118569286	97.1222	37.6838
		chr12	120129022	30826413	116919266	97.3281	37.5965
		chr13	120421639	30513603	116844701	97.0297	37.233
		chr14	124902244	32091164	121424340	97.2155	37.699
		chr15	104043685	25916064	100637545	96.7262	36.6168
		chr16	98207768	24610771	95017360	96.7514	36.7703
		chr17	94987271	24380161	91698270	96.5374	37.5312
		chr18	90702639	22490492	87385995	96.3434	36.411
		chr19	61431566	14947283	58204714	94.7472	35.7062
		chrX	171031299	42595917	163392934	95.5339	36.6267
		chrY	91744698	428628	3237113	3.52839	0.636382
	**N2275-5 (male)**	chr1	195471971	42773251	191756262	98.0991	32.2157
		chr2	182113224	41796762	178079743	97.7852	33.3899
		chr3	160039680	34216330	156088209	97.5309	31.4174
		chr4	156508116	34083252	151901935	97.0569	31.94
		chr5	151834684	32977662	147816603	97.3536	31.9335
		chr6	149736546	32175803	146125878	97.5887	31.6147
		chr7	145441459	31230187	141758821	97.468	31.4788
		chr8	129401213	27915975	125374377	96.8881	31.723
		chr9	124595110	31771111	121060356	97.163	36.5541
		chr10	130694993	28225431	126967744	97.1481	31.7619
		chr11	122082543	27161810	118505197	97.0697	32.5845
		chr12	120129022	26446689	116852273	97.2723	32.2561
		chr13	120421639	25959530	116755958	96.956	31.6942
		chr14	124902244	27229827	121351905	97.1575	31.9253
		chr15	104043685	22085546	100573331	96.6645	31.2376
		chr16	98207768	21020075	94962219	96.6952	31.3912
		chr17	94987271	20959116	91641276	96.4774	32.2509
		chr18	90702639	19264961	87335747	96.288	31.2159
		chr19	61431566	12867744	58169087	94.6892	30.7731
		chrX	171031299	18545674	163145172	95.3891	15.9039
		chrY	91744698	9264207	87964661	95.8798	14.7967
	**N2275-6 (female)**	chr1	195471971	22885787	191588118	98.0131	17.2224
		chr2	182113224	22697165	177897371	97.685	18.0696
		chr3	160039680	18348586	155902252	97.4147	16.8305
		chr4	156508116	18156474	151738517	96.9525	16.9955
		chr5	151834684	17544521	147676707	97.2615	16.972
		chr6	149736546	17205586	145995439	97.5015	16.8882
		chr7	145441459	16555044	141628891	97.3786	16.669
		chr8	129401213	14861987	125227075	96.7743	16.8725

	chr9	124595110	17505558	120954877	97.0784	20.0311
	chr10	130694993	15096412	126840522	97.0508	16.974
	chr11	122082543	14405576	118370015	96.959	17.2589
	chr12	120129022	14170104	116754748	97.1911	17.253
	chr13	120421639	13850648	116588299	96.8167	16.8924
	chr14	124902244	14601699	121243021	97.0703	17.0891
	chr15	104043685	11771507	100480252	96.5751	16.6346
	chr16	98207768	11246789	94876726	96.6082	16.7804
	chr17	94987271	11152849	91559359	96.3912	17.1473
	chr18	90702639	10299069	87245849	96.1889	16.6706
	chr19	61431566	6849886	58117777	94.6057	16.367
	chrX	171031299	19030364	163122644	95.3759	16.36
	chrY	91744698	182762	3750152	4.0876	0.251665
**N2275-8 (female**	chr1	195471971	41275641	191738411	98.09	30.6762
	chr2	182113224	41274542	178079124	97.7848	32.2304
	chr3	160039680	32949832	156083863	97.5282	29.848
	chr4	156508116	32924331	151895484	97.0528	30.4294
	chr5	151834684	31917118	147804075	97.3454	30.4849
	chr6	149736546	31130345	146112435	97.5797	30.1674
	chr7	145441459	30256367	141747153	97.4599	30.0793
	chr8	129401213	27001453	125376007	96.8894	30.2691
	chr9	124595110	31528998	121051534	97.1559	35.569
	chr10	130694993	27253198	126956661	97.1397	30.2617
	chr11	122082543	26389348	118513575	97.0766	31.2265
	chr12	120129022	25655178	116842076	97.2638	30.8598
	chr13	120421639	25169700	116765357	96.9638	30.3131
	chr14	124902244	26354218	121341216	97.1489	30.4484
	chr15	104043685	21333287	100562354	96.654	29.7649
	chr16	98207768	20272193	94953785	96.6866	29.8703
	chr17	94987271	20377886	91634821	96.4706	30.928
	chr18	90702639	18641275	87338392	96.2909	29.8003
	chr19	61431566	12531879	58165625	94.6836	29.5654
	chrX	171031299	33773179	163273027	95.4638	28.6685
	chrY	91744698	344271	5333813	5.81376	0.463477
**N2275-9 (female**	chr1	195471971	46688193	191766801	98.1045	34.8191
	chr2	182113224	46403104	178109567	97.8016	36.4089
	chr3	160039680	37329160	156112899	97.5464	33.9344
	chr4	156508116	37270072	151923696	97.0708	34.5663
	chr5	151834684	36035647	147828716	97.3616	34.5408
	chr6	149736546	35158269	146138293	97.5969	34.1956
	chr7	145441459	34161852	141769605	97.4754	34.0852
	chr8	129401213	30505605	125401048	96.9087	34.3177
	chr9	124595110	35171548	121066245	97.1677	39.8288
	chr10	130694993	30789183	126978083	97.156	34.3077
	chr11	122082543	29679307	118527215	97.0878	35.2386
	chr12	120129022	28982196	116857896	97.277	34.9845
	chr13	120421639	28314919	116790717	96.9848	34.2227
	chr14	124902244	29827897	121360361	97.1643	34.5895
	chr15	104043685	24064096	100578622	96.6696	33.6964
	chr16	98207768	22932265	94965657	96.6987	33.9053
	chr17	94987271	23005786	91646854	96.4833	35.0382
	chr18	90702639	21004469	87350352	96.3041	33.6957
	chr19	61431566	14066509	58173710	94.6968	33.3044
	chrX	171031299	38668283	163307362	95.4839	32.9403

		chrY	91744698	400357	5773156	6.29263	0.540909
	N3215 (female)	chr1	195471971	64952899	191863824	98.15	37.14
		chr2	182113224	89071233	178188360	97.84	36.29
		chr3	160039680	52384468	156190499	97.59	36.47
		chr4	156508116	51301495	152008376	97.12	36.68
		chr5	151834684	50424621	147912494	97.42	37.23
		chr6	149736546	50854520	146216276	97.65	36.39
		chr7	145441459	47382963	141843820	97.53	36.26
		chr8	129401213	42164217	125467656	96.96	36.54
		chr9	124595110	64622811	121126942	97.22	36.8
		chr10	130694993	42682099	127050031	97.21	36.6
		chr11	122082543	40063046	118584324	97.13	36.63
		chr12	120129022	41774839	116918166	97.33	36.88
		chr13	120421639	39277180	116846573	97.03	36.56
		chr14	124902244	42206439	121424279	97.22	37.14
		chr15	104043685	33501732	100637175	96.73	36
		chr16	98207768	31966828	95016683	96.75	36.18
		chr17	94987271	31160031	91697275	96.54	36.47
		chr18	90702639	28981346	87393982	96.35	35.61
		chr19	61431566	19067348	58204073	94.75	34.59
		chrX	171031299	54570738	163390980	95.53	36.1
		chrY	91744698	423623	3525099	3.84	0.56
	N3215-6 (female)	chr1	195471971	43581209	191687850	98.0641	32.9006
		chr2	182113224	59307023	178008155	97.7459	47.1205
		chr3	160039680	35188892	155998121	97.4747	32.4359
		chr4	156508116	34324329	151836432	97.0151	32.3566
		chr5	151834684	34009706	147770211	97.3231	33.0433
		chr6	149736546	33394348	146073936	97.554	32.8382
		chr7	145441459	32209068	141711370	97.4353	32.6897
		chr8	129401213	29004520	125317504	96.8441	33.0302
		chr9	124595110	35937833	121024952	97.1346	42.4889
		chr10	130694993	28907935	126919538	97.1112	32.6226
		chr11	122082543	26932900	118432147	97.0099	32.4718
		chr12	120129022	28393769	116820047	97.2455	34.7814
		chr13	120421639	26699847	116697647	96.9075	32.7033
		chr14	124902244	27800601	121313190	97.1265	32.8642
		chr15	104043685	22891287	100544242	96.6366	32.4467
		chr16	98207768	21295428	94924987	96.6573	32.0013
		chr17	94987271	20534167	91593519	96.4272	31.8972
		chr18	90702639	19905564	87297772	96.2461	32.3508
		chr19	61431566	13229734	58150723	94.6594	31.7571
		chrX	171031299	35975314	163240948	95.4451	31.1026
		chrY	91744698	249855	5239536	5.711	0.406064
	N3215-7 (female)	chr1	195471971	50110557	191865589	98.155	37.8519
		chr2	182113224	47539452	178198388	97.8503	38.3232
		chr3	160039680	39815881	156199228	97.6003	36.7149
		chr4	156508116	39875130	152007635	97.1244	37.5147
		chr5	151834684	38760981	147914861	97.4184	37.5672
		chr6	149736546	37416847	146219129	97.6509	36.9362
		chr7	145441459	36994368	141846004	97.5279	37.1294
		chr8	129401213	32603752	125468722	96.961	37.1838
		chr9	124595110	35939114	121131009	97.2197	41.8305
		chr10	130694993	33129521	127051020	97.2118	37.3723
		chr11	122082543	31946975	118584486	97.1347	38.4141

		chr12	120129022	30704756	116920596	97.3292	37.6111
		chr13	120421639	30214641	116855685	97.0388	37.051
		chr14	124902244	31840415	121427647	97.2181	37.3443
		chr15	104043685	25777625	100639055	96.7277	36.6161
		chr16	98207768	24663333	95018448	96.7525	36.7803
		chr17	94987271	24629938	91699810	96.5391	38.0302
		chr18	90702639	22473981	87395600	96.354	36.5461
		chr19	61431566	15043204	58205107	94.7479	36.1294
		chrX	171031299	41642440	163394823	95.535	36.0064
		chrY	91744698	433035	3590303	3.91336	0.603525
	**B3175 (male)**	chr1	195471971	67377743	191863906	98.15	38.06
		chr2	182113224	83223640	178197687	97.85	37.35
		chr3	160039680	54308831	156204042	97.6	37.35
		chr4	156508116	53200442	152009618	97.13	37.6
		chr5	151834684	52287429	147913883	97.42	38.15
		chr6	149736546	52510972	146217130	97.65	37.36
		chr7	145441459	48943890	141844661	97.53	37
		chr8	129401213	43762160	125472603	96.96	37.47
		chr9	124595110	59835840	121128229	97.22	37.96
		chr10	130694993	44419563	127050822	97.21	37.62
		chr11	122082543	41988112	118594944	97.14	37.98
		chr12	120129022	42800549	116919231	97.33	37.75
		chr13	120421639	40982054	116867279	97.05	37.69
		chr14	124902244	43706874	121426181	97.22	37.96
		chr15	104043685	34951961	100638197	96.73	37.13
		chr16	98207768	33368974	95017456	96.75	37.34
		chr17	94987271	32598063	91698437	96.54	37.74
		chr18	90702639	30272889	87399007	96.36	36.78
		chr19	61431566	20154395	58204682	94.75	36.18
		chrX	171031299	29010732	163384427	95.53	18.98
		chrY	91744698	14233741	88068871	95.99	20.13
	**N3221 (female)**	chr1	195471971	51063028	191866624	98.1556	38.5092
		chr2	182113224	47868779	178196796	97.8495	38.5545
		chr3	160039680	40699519	156198843	97.6001	37.4635
		chr4	156508116	40240145	152010092	97.126	37.8
		chr5	151834684	39226703	147914372	97.418	37.9729
		chr6	149736546	38069438	146218912	97.6508	37.5118
		chr7	145441459	37258367	141845683	97.5277	37.4126
		chr8	129401213	33063129	125470235	96.9622	37.6483
		chr9	124595110	35066893	121129819	97.2188	40.8969
		chr10	130694993	33511443	127050631	97.2116	37.7182
		chr11	122082543	31672141	118587468	97.1371	38.0354
		chr12	120129022	31157297	116920119	97.3288	38.1268
		chr13	120421639	30585857	116854402	97.0377	37.4401
		chr14	124902244	32433849	121427059	97.2177	38.0528
		chr15	104043685	26132807	100639778	96.7284	37.0479
		chr16	98207768	25085975	95018274	96.7523	37.4263
		chr17	94987271	24613704	91699544	96.5388	37.9531
		chr18	90702639	22675590	87396932	96.3554	36.8097
		chr19	61431566	15035008	58205201	94.748	36.0352
		chrX	171031299	42651736	163396245	95.5359	36.8068
		chrY	91744698	380071	3696336	4.02894	0.523357
	**N3221-1 (male)**	chr1	195471971	42441437	191673869	98.057	32.1506
		chr2	182113224	58997928	177987521	97.7345	46.9938

		chr3	160039680	34159169	155979939	97.4633	31.5932
		chr4	156508116	33581857	151826839	97.0089	31.7633
		chr5	151834684	33188589	147755912	97.3137	32.3564
		chr6	149736546	32492549	146067913	97.5499	32.0636
		chr7	145441459	31605272	141699289	97.427	32.1867
		chr8	129401213	28324898	125300759	96.8312	32.3677
		chr9	124595110	35754960	121019596	97.1303	42.3876
		chr10	130694993	28136988	126901591	97.0975	31.8633
		chr11	122082543	26508283	118418891	96.999	32.0734
		chr12	120129022	27803166	116808161	97.2356	34.1688
		chr13	120421639	26033588	116678950	96.892	31.9963
		chr14	124902244	27069314	121304224	97.1193	32.1044
		chr15	104043685	22330429	100530643	96.6235	31.7605
		chr16	98207768	20666556	94917216	96.6494	31.1625
		chr17	94987271	20131110	91586640	96.4199	31.3769
		chr18	90702639	19406507	87289171	96.2366	31.6511
		chr19	61431566	12973462	58146786	94.6529	31.2547
		chrX	171031299	18247960	162996247	95.302	15.8277
		chrY	91744698	8312604	87816455	95.7183	13.5594
	**N3221-3 (male)**	chr1	195471971	49774228	191863415	98.1539	37.3589
		chr2	182113224	47067337	178185395	97.8432	37.8074
		chr3	160039680	40104600	156182317	97.5897	36.7324
		chr4	156508116	39796642	152000077	97.1196	37.2063
		chr5	151834684	39005024	147913489	97.4175	37.6322
		chr6	149736546	37557922	146215581	97.6486	36.7898
		chr7	145441459	36447615	141844791	97.5271	36.6176
		chr8	129401213	32618882	125451696	96.9479	36.9308
		chr9	124595110	35418826	121126057	97.2157	41.1771
		chr10	130694993	33095098	127048560	97.21	37.1242
		chr11	122082543	31570642	118567314	97.1206	37.7648
		chr12	120129022	30801690	116919395	97.3282	37.4842
		chr13	120421639	30476103	116844905	97.0298	37.0961
		chr14	124902244	32005508	121423923	97.2152	37.509
		chr15	104043685	25927432	100639172	96.7278	36.543
		chr16	98207768	24572547	95017320	96.7513	36.6306
		chr17	94987271	24362959	91698624	96.5378	37.3995
		chr18	90702639	22492746	87386149	96.3436	36.3246
		chr19	61431566	15001964	58204704	94.7472	35.7396
		chrX	171031299	21986674	163382216	95.5277	18.8307
		chrY	91744698	11913313	88059438	95.9831	19.0075
	**N3221-7 (male)**	chr1	195471971	50252655	191862691	98.1536	37.7283
		chr2	182113224	47384650	178186432	97.8438	38.0738
		chr3	160039680	40530507	156183191	97.5903	37.1323
		chr4	156508116	40095937	152001387	97.1205	37.502
		chr5	151834684	39244336	147913587	97.4175	37.881
		chr6	149736546	37895345	146215105	97.6482	37.1321
		chr7	145441459	36670674	141844337	97.5268	36.8726
		chr8	129401213	32852290	125454315	96.9499	37.2096
		chr9	124595110	35634669	121125402	97.2152	41.445
		chr10	130694993	33363662	127048290	97.2098	37.4376
		chr11	122082543	31688998	118568798	97.1218	37.9243
		chr12	120129022	31027105	116918263	97.3272	37.77
		chr13	120421639	30703535	116852880	97.0364	37.3874
		chr14	124902244	32309200	121424088	97.2153	37.8789

		chr15	104043685	26143778	100638115	96.7268	36.8607
		chr16	98207768	24799824	95017202	96.7512	36.98
		chr17	94987271	24512090	91698378	96.5375	37.6506
		chr18	90702639	22711200	87387627	96.3452	36.6905
		chr19	61431566	15098682	58204248	94.7465	35.9879
		chrX	171031299	22213204	163381561	95.5273	19.0288
		chrY	91744698	11875589	88059378	95.9831	18.9489
	**N3221-8 (female)**	chr1	195471971	46041646	191684873	98.0626	34.8251
		chr2	182113224	63434883	178000456	97.7416	50.4683
		chr3	160039680	37245654	155991899	97.4708	34.3963
		chr4	156508116	36273660	151833915	97.0134	34.2594
		chr5	151834684	35949783	147765684	97.3201	34.9966
		chr6	149736546	35322836	146070008	97.5513	34.7968
		chr7	145441459	34015998	141706365	97.4319	34.5907
		chr8	129401213	30747370	125320505	96.8465	35.085
		chr9	124595110	38376215	121024370	97.1341	45.4466
		chr10	130694993	30623497	126913675	97.1068	34.6279
		chr11	122082543	28559616	118430878	97.0089	34.5047
		chr12	120129022	37751872	116836803	97.2594	46.3552
		chr13	120421639	28317491	116685828	96.8977	34.7525
		chr14	124902244	29412421	121310387	97.1243	34.8338
		chr15	104043685	24248391	100537548	96.6301	34.4342
		chr16	98207768	22571325	94924621	96.6569	33.9831
		chr17	94987271	21741614	91587262	96.4206	33.8359
		chr18	90702639	21092187	87300218	96.2488	34.3477
		chr19	61431566	14027695	58149073	94.6567	33.7437
		chrX	171031299	37696374	163235682	95.442	32.647
		chrY	91744698	276959	5700076	6.21298	0.450161

Gene	ENTREZ_ID	David Gene Name	KEGG	GO_BP	GO_MF	GO_CC
EXD1	241624	exonuclease 3'-5' domain containing 1(Exd1)	#N/A	nucleobase-containing compound metabolic process, gene silencing by RNA, piRNA metabolic process, meiotic cell cycle,	nucleic acid binding, RNA binding, 3'-5' exonuclease activity, protein homodimerization activity,	cytoplasm, P granule, PET complex,
MAML1	103806	mastermind like 1 (Drosophila)(Maml1)	Notch signaling pathway,	transcription, DNA-templated, regulation of transcription, DNA-templated, protein phosphorylation, Notch signaling pathway, positive regulation of transcription of Notch receptor target, positive regulation of myotube differentiation, myoblast differentiation, positive regulation of transcription from RNA polymerase II promoter, positive regulation of muscle cell differentiation, atrioventricular node cell development,	transcription coactivator activity, protein kinase binding, peptide antigen binding,	MAML1-RBP-Jkappa- ICN1 complex, nucleus, nucleoplasm, nuclear speck, intracellular membrane-bounded organelle,
GRIK2	14806	glutamate receptor, ionotropic, kainate 2 (beta 2)(Grik2)	Neuroactive ligand-receptor interaction, Glutamatergic synapse,	behavioral fear response, transport, ion transport, cellular calcium ion homeostasis, intracellular protein transport, chemical synaptic transmission, neuronal action potential, synaptic transmission, glutamatergic, regulation of membrane potential, receptor clustering, negative regulation of neuron apoptotic process, positive regulation of neuron apoptotic process, regulation of JNK cascade, regulation of long-term neuronal synaptic plasticity, regulation of short-term neuronal synaptic plasticity, modulation of synaptic transmission, positive regulation of synaptic transmission, neuron apoptotic process, negative regulation of synaptic transmission, glutamatergic, excitatory postsynaptic potential, inhibitory postsynaptic potential,	receptor activity, ionotropic glutamate receptor activity, ion channel activity, extracellular-glutamate-gated ion channel activity, protein binding, glutamate receptor activity, kainate selective glutamate receptor activity, PDZ domain binding, ubiquitin conjugating enzyme binding, ubiquitin protein ligase binding, identical protein binding, protein homodimerization activity,	plasma membrane, integral component of plasma membrane, ionotropic glutamate receptor complex, postsynaptic density, membrane, integral component of membrane, cell junction, axon, dendrite, dendrite cytoplasm, kainate selective glutamate receptor complex, presynaptic membrane, neuronal cell body, terminal bouton, perikaryon, synapse, postsynaptic membrane,
MTFR1L	76824	mitochondrial fission regulator 1-	#N/A	mitochondrial fission, aerobic respiration,	#N/A	mitochondrion,
GNAI1	14677	guanine nucleotide binding protein (G protein), alpha inhibiting 1(Gnai1)	Rap1 signaling pathway, cGMP-PKG signaling pathway, cAMP signaling pathway, Chemokine signaling pathway, Sphingolipid signaling pathway, Adrenergic signaling in cardiomyocytes, Axon guidance, Gap junction, Platelet activation, Leukocyte transendothelial migration, Circadian entrainment, Retrograde endocannabinoid signaling, Glutamatergic synapse, Cholinergic synapse, Serotonergic synapse, GABAergic synapse, Dopaminergic synapse, Long-term depression, Progesterone-mediated oocyte maturation, Estrogen signaling pathway, Melanogenesis, Oxytocin signaling pathway, Regulation of lipolysis in adipocytes, Renin secretion, Gastric acid secretion, Parkinson's disease, Cocaine addiction, Morphine addiction, Alcoholism, Pertussis,	cell cycle, signal transduction, G-protein coupled receptor signaling pathway, adenylate cyclase-modulating G-protein coupled receptor signaling pathway, regulation of cAMP-mediated signaling, negative regulation of synaptic transmission, cell division, cellular response to forskolin,	nucleotide binding, magnesium ion binding, G-protein coupled receptor binding, GTPase activity, signal transducer activity, protein binding, GTP binding, guanyl nucleotide binding, GDP binding, G-protein beta/gamma-subunit complex binding, G-protein coupled serotonin receptor binding, GTPase activating protein binding, metal ion binding,	intracellular, nucleus, cytoplasm, lysosomal membrane, centrosome, heterotrimeric G-protein complex, cytoskeleton, plasma membrane, membrane, midbody, protein complex, membrane raft, extracellular exosome,
PRSS43	272643	protease, serine 43(Prss43)	#N/A	germ cell development, spermatogenesis,	serine-type endopeptidase activity,	integral component of membrane, anchored component of plasma membrane,
TRAK2	70827	trafficking protein, kinesin binding 2(Trak2)	Metabolic pathways, GABAergic synapse,	regulation of transcription from RNA polymerase II promoter, protein O-linked glycosylation, protein targeting,	receptor binding, enzyme binding, GABA receptor binding,	nucleus, cytoplasm, mitochondrion, plasma membrane,

PURA	19290	purine rich element binding protein A(Pura)	#N/A	DNA unwinding involved in DNA replication, transcription, DNA-templated, regulation of transcription, DNA-templated, apoptotic process, mitotic cell cycle checkpoint, nervous system development, cell proliferation, positive regulation of cell proliferation, cell differentiation, regulation of cell proliferation, negative regulation of transcription, DNA-templated,	translation repressor activity, nucleic acid binding, DNA binding, double-stranded DNA binding, double-stranded telomeric DNA binding, single-stranded DNA binding, transcription factor activity, sequence-specific DNA binding, RNA binding, protein binding, transcription factor binding, purine-rich negative regulatory element binding, poly(A) RNA binding, SMAD binding,	nucleus, DNA replication factor A complex, cytoplasm, dendrite, neuronal cell body,
ADAMTSL3	269959	ADAMTS-like 3(Adamtsl3)	#N/A	#N/A	peptidase activity, metallopeptidase activity, zinc ion binding,	proteinaceous extracellular matrix,
ADAM24	13526	a disintegrin and metallopeptidase domain 24 (testase 1)(Adam24)	#N/A	proteolysis, integrin-mediated signaling pathway, multicellular organism development, spermatogenesis, cell differentiation, prevention of polyspermy,	metalloendopeptidase activity, integrin binding, peptidase activity, metallopeptidase activity, hydrolase activity, metal ion binding,	membrane, integral component of membrane,
CREG1	433375	cellular repressor of E1A-stimulated genes 1(Creg1)	#N/A	regulation of transcription, DNA-templated, regulation of growth, oxidation-reduction process,	transcription factor binding, FMN binding, oxidoreductase activity,	extracellular region, extracellular space, transcription factor complex, extracellular exosome,
HMCN2	665700	hemicentin 2(Hmcn2)	#N/A	response to stimulus,	calcium ion binding,	extracellular region, proteinaceous extracellular matrix, basement membrane, cell cortex, cell junction,
RAB11FIP3	215445	RAB11 family interacting protein 3 (class II)(Rab11fip3)	Endocytosis,	cytokinesis, transport, cell cycle, vesicle-mediated transport, endocytic recycling, cell division, protein localization to cilium, negative regulation of adiponectin secretion,	calcium ion binding, protein binding, Rab GTPase binding, ADP-ribosylation factor binding, protein homodimerization activity, metal ion binding, dynein light intermediate chain binding,	nucleoplasm, cytoplasm, endosome, centrosome, microtubule organizing center, cytoskeleton, membrane, midbody, cleavage furrow, intracellular membrane-bounded organelle, intercellular bridge, recycling endosome, recycling endosome membrane,
MUC4	140474	mucin 4(Muc4)	#N/A	negative regulation of cell-matrix adhesion, hematopoietic progenitor cell differentiation, negative regulation of T cell mediated cytotoxicity directed against tumor cell target, cell adhesion, cell-matrix adhesion, regulation of receptor activity, negative regulation of cell-cell adhesion, negative regulation of apoptotic process,	ErbB-2 class receptor binding, protein complex binding,	extracellular region, extracellular space, cytoplasm, endoplasmic reticulum, membrane, integral component of membrane, apical plasma membrane, microvillus membrane, protein complex,
CFAP44	212517	cilia and flagella associated protein 44(Cfap44)	#N/A	cilium-dependent cell motility,	microtubule binding,	#N/A
HERC6	67138	hect domain and RLD 6(Herc6)	#N/A	hematopoietic progenitor cell differentiation, immune system process, protein ubiquitination, protein ubiquitination involved in ubiquitin-dependent protein catabolic process, innate immune response,	ubiquitin-protein transferase activity, transferase activity, cyclin binding, ubiquitin protein ligase activity,	nucleus, cytoplasm, cytosol,
EGLN1	112405	egl-9 family hypoxia-inducible factor 1(Egln1)	HIF-1 signaling pathway, Pathways in cancer, Renal cell carcinoma,	response to hypoxia, cellular iron ion homeostasis, peptidyl-proline hydroxylation to 4-hydroxy-L-proline, negative regulation of cAMP catabolic process, oxygen homeostasis, positive regulation of apoptotic process, negative regulation of sequence-specific DNA binding transcription factor activity, regulation of angiogenesis, positive regulation of transcription from RNA polymerase II promoter, negative regulation of cyclic-nucleotide phosphodiesterase activity, cardiac muscle tissue morphogenesis, oxidation-reduction process, heart trabecula formation, ventricular septum morphogenesis, labyrinthine layer development, response to nitric oxide, regulation of neuron death, positive regulation of neuron death, negative regulation of CAMKK-AMPK signaling cascade,	iron ion binding, oxidoreductase activity, oxidoreductase activity, acting on paired donors, with incorporation or reduction of molecular oxygen, oxidoreductase activity, acting on paired donors, with incorporation or reduction of molecular oxygen, 2-oxoglutarate as one donor, and incorporation of one atom each of oxygen into both donors, enzyme binding, L-ascorbic acid binding, peptidyl-proline dioxygenase activity, peptidyl-proline 4-dioxygenase activity, metal ion binding, dioxygenase activity,	nucleus, cytoplasm, cytosol,

ANXA3	11745	annexin A3(Anxa3)	#N/A	phagocytosis, positive regulation of endothelial cell migration, hippocampus development, organ regeneration, defense response to bacterium, neutrophil degranulation, positive regulation of angiogenesis, positive regulation of DNA metabolic process, positive regulation of sequence-specific DNA binding transcription factor activity, response to glucocorticoid, response to growth factor,	phospholipase inhibitor activity, calcium ion binding, calcium-dependent phospholipid binding, phospholipase A2 inhibitor activity, calcium-dependent protein binding,	cytoplasm, plasma membrane, membrane, axon, dendrite, phagocytic vesicle membrane, specific granule, neuronal cell body, extracellular exosome,
GLCE	93683	glucuronyl C5-epimerase(Glce)	Glycosaminoglycan biosynthesis - heparan sulfate / heparin, Metabolic pathways,	glycosaminoglycan biosynthetic process, heparan sulfate proteoglycan biosynthetic process, heparin biosynthetic process,	isomerase activity, racemase and epimerase activity, acting on carbohydrates and derivatives, heparosan-N-sulfate-glucuronate 5-epimerase activity, UDP-glucuronate 5'-	Golgi membrane, Golgi apparatus, membrane, integral component of membrane,
GM35315	102638847	predicted gene, 35315(Gm35315)	#N/A	#N/A	#N/A	#N/A
OLFR1467	258686	olfactory receptor 1467(Olfr1467)	Olfactory transduction,	G-protein coupled receptor signaling pathway, sensory perception of smell,	G-protein coupled receptor activity, olfactory receptor activity, odorant	plasma membrane, integral component of membrane,
CDC7	12545	cell division cycle 7 (S. cerevisiae)(Cdc7)	Cell cycle,	double-strand break repair via break-induced replication, DNA replication initiation, protein phosphorylation, phagocytosis, cell cycle, positive regulation of cell proliferation, regulation of cell shape, positive regulation of nuclear cell cycle DNA replication, positive regulation of G2/M transition of mitotic cell cycle, phosphorylation, peptidyl-serine phosphorylation, cell cycle phase transition, cell division,	nucleotide binding, protein kinase activity, protein serine/threonine kinase activity, ATP binding, kinase activity, transferase activity, metal ion binding,	nucleus, nucleoplasm, cytoplasm, microtubule cytoskeleton, intercellular bridge,
PTPN13	19249	protein tyrosine phosphatase, non-receptor type 13(Ptpn13)	#N/A	protein dephosphorylation, regulation of phosphatidylinositol 3-kinase signaling, dephosphorylation, peptidyl-tyrosine dephosphorylation, bicellular tight junction assembly,	phosphoprotein phosphatase activity, protein tyrosine phosphatase activity, protein binding, hydrolase activity, phosphatase activity, phosphatidylinositol 3-kinase regulatory	nucleus, cytoplasm, cytoskeleton, plasma membrane, lamellipodium, midbody, cell projection, neuron projection, cell body, extracellular exosome,
MEIS2	17536	Meis homeobox 2(Meis2)	#N/A	eye development, transcription, DNA-templated, regulation of transcription, DNA-templated, multicellular organism development, visual learning, response to mechanical stimulus, pancreas development, negative regulation of myeloid cell differentiation, positive regulation of transcription from RNA polymerase II promoter, response to growth factor,	RNA polymerase II core promoter proximal region sequence-specific DNA binding, transcriptional activator activity, RNA polymerase II core promoter proximal region sequence-specific binding, DNA binding, transcription cofactor activity, transcription factor binding, sequence-specific DNA binding,	nucleus, cytoplasm, perinuclear region of cytoplasm,
GM5724	435927	predicted gene 5724(Gm5724)	#N/A	bile acid metabolic process, sodium-independent organic anion transport,	bile acid transmembrane transporter activity, sodium-independent organic anion transmembrane transporter	integral component of plasma membrane,
PTK7	71461	PTK7 protein tyrosine kinase 7(Ptk7)	#N/A	establishment of planar polarity, neural tube closure, ventricular septum development, axis elongation, protein phosphorylation, cell adhesion, heart development, positive regulation of neuron projection development, Wnt signaling pathway, cell migration, actin cytoskeleton reorganization, wound healing, establishment of epithelial cell apical/basal polarity, convergent extension, canonical Wnt signaling pathway, lung-associated mesenchyme development, coronary vasculature development, cellular response to retinoic acid, cochlea morphogenesis, planar cell polarity pathway involved in neural tube closure,	protein kinase activity, transmembrane receptor protein tyrosine kinase activity, ATP binding, kinase activity, coreceptor activity involved in Wnt signaling pathway, planar cell polarity pathway,	plasma membrane, integral component of plasma membrane, cell-cell junction, focal adhesion, membrane, integral component of membrane, cell junction,

NACA	17938	nascent polypeptide-associated complex alpha polypeptide(Naca)	#N/A	cardiac ventricle development, transcription, DNA-templated, regulation of transcription, DNA-templated, transport, negative regulation of striated muscle cell apoptotic process, protein transport, skeletal muscle tissue regeneration, positive regulation of skeletal muscle tissue growth, regulation of skeletal muscle fiber development, myoblast migration, heart trabecula morphogenesis, negative regulation of transcription from RNA polymerase II promoter involved in heart development, positive regulation of transcription from RNA polymerase II promoter involved in heart development, positive regulation of cell proliferation involved in heart morphogenesis,	DNA binding, transcription coactivator activity, TBP-class protein binding,	nucleus, cytoplasm, extracellular exosome,
NEDD4	17999	neural precursor cell expressed, developmentally down-regulated 4(Nedd4)	Ubiquitin mediated proteolysis, Endocytosis, Epstein-Barr virus infection,	negative regulation of transcription from RNA polymerase II promoter, adaptive immune response, outflow tract morphogenesis, endocardial cushion development, protein monoubiquitination, protein targeting to lysosome, nervous system development, neuromuscular junction development, negative regulation of sodium ion transport, negative regulation of transcription from RNA polymerase II promoter in response to UV-induced DNA damage, positive regulation of phosphatidylinositol 3-kinase signaling, protein ubiquitination, transmission of virus, negative regulation of vascular endothelial growth factor receptor signaling pathway, neuron projection development, receptor internalization, receptor catabolic process, T cell activation, regulation of membrane potential, protein ubiquitination involved in ubiquitin-dependent protein catabolic process, glucocorticoid receptor signaling pathway, ubiquitin-dependent protein catabolic process via the multivesicular body sorting pathway, development involved in symbiotic interaction, positive regulation of protein catabolic process, positive regulation of nucleocytoplasmic transport, blood vessel morphogenesis, regulation of dendrite morphogenesis, regulation of synapse organization, progesterone receptor signaling pathway, protein K63-linked ubiquitination, regulation of potassium ion transmembrane transporter activity, negative regulation of sodium ion transmembrane transporter activity,	ubiquitin-protein transferase activity, protein binding, protein C-terminus binding, transferase activity, ligase activity, sodium channel inhibitor activity, ionotropic glutamate receptor binding, phosphoserine binding, phosphothreonine binding, ubiquitin protein ligase activity, proline-rich region binding,	ubiquitin ligase complex, chromatin, nucleus, cytoplasm, Golgi apparatus, cytosol, plasma membrane, microvillus, cell cortex, membrane, membrane raft, perinuclear region of cytoplasm, extracellular exosome,
POU2AF1	18985	POU domain, class 2, associating factor 1(Pou2af1)	#N/A	transcription, DNA-templated, regulation of transcription, DNA-templated,	DNA binding, protein binding,	nucleus,
VMN2R88	669149	vomeronasal 2, receptor 88(Vmn2r88)	#N/A	#N/A	G-protein coupled receptor activity,	plasma membrane, integral component of membrane,
GM3667	100042100	predicted gene 3667(Gm3667)	#N/A	#N/A	#N/A	#N/A
ZFP131	72465	zinc finger protein 131(Zfp131)	#N/A	transcription, DNA-templated, regulation of transcription, DNA-templated, regulation of transcription from RNA polymerase II promoter,	RNA polymerase II transcription factor activity, sequence-specific DNA binding, nucleic acid binding, DNA binding, transcription factor activity, sequence-specific DNA binding, metal ion binding,	nucleus,
TPP2	22019	tripeptidyl peptidase II(Tpp2)	#N/A	proteolysis,	aminopeptidase activity, serine-type endopeptidase activity, peptidase activity, serine-type peptidase activity, tripeptidyl-peptidase activity, hydrolase activity, peptide binding,	nucleus, cytoplasm,

RAPGEF4	56508	Rap guanine nucleotide exchange factor (GEF) 4(Rapgef4)	Rap1 signaling pathway, cAMP signaling pathway, Adrenergic signaling in cardiomyocytes, Leukocyte transendothelial migration, Insulin secretion,	exocytosis, signal transduction, small GTPase mediated signal transduction, positive regulation of smooth muscle cell migration, calcium ion regulated exocytosis, regulation of exocytosis, cAMP-mediated signaling, insulin secretion, intracellular signal transduction, positive regulation of GTPase activity, positive regulation of protein secretion, regulation of dendrite development, negative regulation of synaptic transmission, positive regulation of neuronal action potential,	nucleotide binding, guanyl-nucleotide exchange factor activity, Ras guanyl-nucleotide exchange factor activity, protein binding, Ras GTPase binding, cAMP binding, protein complex binding,	photoreceptor outer segment, photoreceptor inner segment, intracellular, cytoplasm, cytosol, plasma membrane, brush border, cilium, membrane, basolateral plasma membrane, apical plasma membrane, axon, dendrite, growth cone, neuronal cell body, dendritic spine, protein complex, cone cell pedicle, excitatory synapse,

	A	B	C	D	E	F	G
1	**Category**	**Term**	**Genes Count**		**Percentage**	**P-value**	**Q-value**
2	GOTERM_BP_DIRECT	negative regulation of synaptic transmission		2	5.9	0.02	1.00
3	GOTERM_BP_DIRECT	response to growth factor		2	5.9	0.02	1.00
4	GOTERM_BP_DIRECT	phagocytosis		2	5.9	0.08	1.00
5	GOTERM_CC_DIRECT	midbody		3	8.8	0.02	0.92
6	GOTERM_CC_DIRECT	dendrite		4	11.8	0.04	0.92
7	GOTERM_CC_DIRECT	neuronal cell body		4	11.8	0.05	0.92
8	GOTERM_CC_DIRECT	cytoplasm		16	47.1	0.05	0.92
9	GOTERM_CC_DIRECT	intercellular bridge		2	5.9	0.07	1.00
10	GOTERM_CC_DIRECT	nucleus		14	41.2	0.10	1.00
11	INTERPRO	Immunoglobulin I-set		3	8.8	0.02	1.00
12	INTERPRO	HECT		2	5.9	0.04	1.00
13	INTERPRO	Epidermal growth factor-like domain		3	8.8	0.05	1.00
14	INTERPRO	Immunoglobulin subtype 2		3	8.8	0.05	1.00
15	SMART	HECTc		2	5.9	0.05	1.00
16	SMART	IGc2		3	8.8	0.08	1.00
17	UP_KEYWORDS	EGF-like domain		3	8.8	0.04	1.00
18	UP_KEYWORDS	Activator		4	11.8	0.06	1.00
19	UP_KEYWORDS	Cytoplasm		11	32.4	0.09	1.00
20							
21							
22							
